# Origin of the Proton-transfer Step in the Cofactor-free (1*H*)-3-Hydroxy-4-oxoquinaldine 2,4-Dioxygenase: EFFECT OF THE BASICITY OF AN ACTIVE SITE HIS RESIDUE*[Fn FN2]

**DOI:** 10.1074/jbc.M113.543033

**Published:** 2014-01-30

**Authors:** Aitor Hernandez-Ortega, Matthew G. Quesne, Soi Bui, Dominic P. H. M. Heuts, Roberto A. Steiner, Derren J. Heyes, Sam P. de Visser, Nigel S. Scrutton

**Affiliations:** From the ‡ Manchester Institute of Biotechnology, University of Manchester, Manchester M1 7DN and; the § Randall Division of Cell and Molecular Biophysics, King's College London, London SE1 1UL, United Kingdom

**Keywords:** Enzyme Kinetics, Enzyme Mechanisms, Isotope Effects, Molecular Dynamics, Site-directed Mutagenesis, DFT Calculations, Quantum Mechanics/Molecular Mechanics

## Abstract

Dioxygenases catalyze a diverse range of chemical reactions that involve the incorporation of oxygen into a substrate and typically use a transition metal or organic cofactor for reaction. Bacterial (1*H*)-3-hydroxy-4-oxoquinaldine 2,4-dioxygenase (HOD) belongs to a class of oxygenases able to catalyze this energetically unfavorable reaction without any cofactor. In the quinaldine metabolic pathway, HOD breaks down its natural *N*-heteroaromatic substrate using a mechanism that is still incompletely understood. Experimental and computational approaches were combined to study the initial step of the catalytic cycle. We have investigated the role of the active site His-251/Asp-126 dyad, proposed to be involved in substrate hydroxyl group deprotonation, a critical requirement for subsequent oxygen reaction. The pH profiles obtained under steady-state conditions for the H251A and D126A variants show a strong pH effect on their *k*_cat_ and *k*_cat_/*K_m_* constants, with a decrease in *k*_cat_/*K_m_* of 5500- and 9-fold at pH 10.5, respectively. Substrate deprotonation studies under transient-state conditions show that this step is not rate-limiting and yield a p*K_a_* value of ∼7.2 for WT HOD. A large solvent isotope effect was found, and the p*K_a_* value was shifted to ∼8.3 in D_2_O. Crystallographic and computational studies reveal that the mutations have a minor effect on substrate positioning. Computational work shows that both His-251 and Asp-126 are essential for the proton transfer driving force of the initial reaction. This multidisciplinary study offers unambiguous support to the view that substrate deprotonation, driven by the His/Asp dyad, is an essential requirement for its activation.

## Teaser

**Background:** The mechanism of cofactor-free dioxygenases has not been clearly elucidated.

**Results:** Mutation of the His/Asp dyad in (1*H*)-3-hydroxy-4-oxoquinaldine 2,4-dioxygenase strongly affects substrate deprotonation and overall catalysis.

**Conclusion:** Base mechanism is demonstrated where His-251 acts as catalytic base and Asp-126 modulates basicity.

**Significance:** Many dioxygenases activate their substrates via deprotonation, which is an essential step for later reaction with oxygen.

## Introduction

Dioxygenases catalyze the incorporation of molecular oxygen into organic compounds in a wide range of biological processes. In humans, they are involved in the pathogenesis of autoimmune disease as well as in the maintenance of self-tolerance (1). Moreover, they play a key role in the immunomodulatory effects on several types of cells (2), are essential in the biosynthesis of collagens (3), and regulate the expression of genes involved in the hypoxia response (4). In addition, dioxygenases maintain carotenoid homeostasis in tissues (5), participate in the metabolism of cysteine and other amino acids (6), and repair DNA alkylation damage (7). Finally, dioxygenases are important in the biosynthesis of signaling compounds (abscisic acid, gibberellins, and ethylene) and secondary metabolites (flavonoids and alkaloids) in plants (8). Bacterial aromatic dioxygenases catalyze enantiospecific reactions, which make them attractive for the production of industrially chiral chemicals and also for bioremediation of xenobiotic contaminated soils (9, 10).

Because of the difficult chemistry and high energetic costs associated with oxygen activation, the majority of dioxygenases use either a metal ion (typically heme and non-heme iron and manganese) or an organic (flavin) cofactor for catalysis. A common problem of oxidases and oxygenases is that a direct reaction of oxygen (triplet state) with either a cofactor or an organic molecule (singlet state) to form singlet products violates the Wigner spin-conservation rule. For that reason, ground-state oxygen has to be activated by changing its spin state. Flavins and flavoproteins react with oxygen by transferring one electron from the reduced cofactor leading to the flavin semiquinone-superoxide radical pair, which after spin conversion may recombine to form a flavin hydroperoxide intermediate (11). Alternatively, many dioxygenases use a metal cofactor and are able to mediate the triplet to singlet conversion by producing metal-bound activated oxygen species (12). However, some oxygenases have been identified that can catalyze oxygen incorporation in the absence of either a metal ion or an organic cofactor (13). The reaction mechanism of these so-called cofactor-free or cofactor-independent dioxygenases is still poorly understood, and it is unclear how these cofactor-free dioxygenases overcome the significant activation barrier for oxygen chemistry. They are fascinating to study mechanistically as they are able to activate the spin-forbidden reaction between dioxygen and their substrates without the requirement of any kind of cofactor. Hence, a detailed characterization of all reaction steps in the catalytic cycle will be important for understanding how these enzymes work.

**SCHEME 1.**
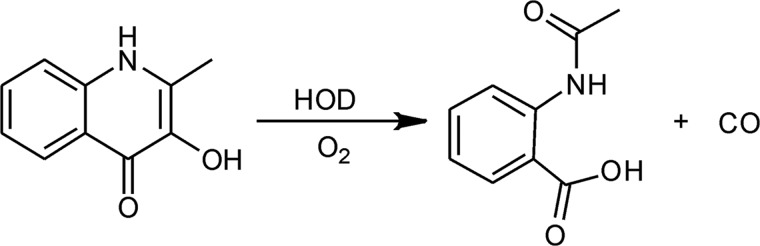
**Overall reaction catalyzed by HOD.**

*Arthrobacter nitroguajacolicus* Rü61a (1*H*)-3-hydroxy-4-oxoquinaldine 2,4-dioxygenase (HOD)^5^ and *Pseudomonas putida* 33/1 (1*H*)-3-hydroxy-4-oxoquinoline 2,4-dioxygenase (QDO) were among the first cofactor-independent oxygenases discovered and described. They are ∼31-kDa monomeric enzymes that catalyze an O_2_-dependent *N*-heteroaromatic ring cleavage reaction with concomitant release of carbon monoxide (Scheme 1) (14). This reaction is chemically identical to the one catalyzed by the copper-dependent quercetin dioxygenase (15). Neither HOD nor QDO is related in sequence to either quercetin dioxygenase or other known oxygenases (16). However, they share a 37% sequence identity; furthermore, their crystal structures are similar, indicating that they constitute a separate dioxygenase family (17). Remarkably, HOD and QDO are members of the α/β-hydrolase fold superfamily (16, 17). This large family is mainly constituted by hydrolases with diverse catalytic functions, although they do not typically catalyze oxygenation reactions (18, 19). The only α/β-hydrolase fold enzyme known to be involved in an oxygen incorporation reaction is *Renilla reniformis* luciferase monooxygenase whose reaction mechanism has not yet been elucidated (20).

Highly conserved catalytic residues among α/β-hydrolases form a triad consisting of nucleophile/histidine/acid set of residues (18, 19). The corresponding catalytic triads in HOD and QDO are Ser-101/His-251/Asp-126 and Ser-95/His-244/Asp-120, respectively (16). Site-directed mutagenesis studies found the His/Asp dyad important for catalysis, whereas S101A replacement in HOD impacted negatively only on *K_m_*, suggesting that this member of the triad has a role in the stabilization of the organic reactant but it is not strictly required for catalysis. Thus, cofactor-independent α/β-hydrolase fold dioxygenases have been proposed to follow the principles of general base catalysis and not a more classical nucleophilic mechanism (17).

In this work, we have provided a detailed characterization of the first reaction step during HOD catalysis. We have explored the role of two conserved active site residues (His-251 and Asp-126) and have gained information about substrate binding and catalysis by using a multidisciplinary approach that combines experimental and computational tools. This has included site-directed mutagenesis followed by steady- and transient-state kinetics to quantify the involvement of these residues in catalysis as well as crystallographic analysis of the H251A variant. Computational studies have been used to explain the reaction mechanism in energetic terms. Our data show that this cofactor-independent dioxygenase employs a simple His/Asp dyad in the active site to assist in deprotonation of the bound substrate. Our work is fully consistent with the proposal (17) that His-251 is a catalytic base, required to activate the substrate through deprotonation, which then facilitates subsequent reaction with molecular oxygen.

## EXPERIMENTAL PROCEDURES

### 

#### 

##### Materials

All chemicals were obtained from Sigma unless otherwise stated.

##### DNA Techniques

HOD cDNA, carrying a C69S mutation to prevent partial oxidative protein dimerization (21), was cloned into the pQE30 (Qiagen) vector. This mutation has no effect on HOD activity (22), and hereafter we will refer to HOD C69S as WT HOD. The corresponding pQE30-wtHOD construct was used to transform *Escherichia coli* M15 (pREP4) strain (Qiagen). The latter was used to inoculate starter cultures for WT HOD expression. Site-directed mutagenesis was performed following the QuikChange^TM^ site-directed mutagenesis kit (Stratagene) protocol, using pQE30-wtHOD as template, and the following oligonucleotides (direct sequences) bearing mutations (underlined) at the corresponding triplets (boldface) were used as primers: H251A, 5′-GGGCGGGCCGACC**GCC**TTCCCCGCCATCGACG-3′; D126A, 5′-CGAGGCATCATCATG**GCC**TGGCTAATGTGGGC-3′. Mutations were confirmed by sequence analysis.

##### Expression and Purification of HOD Samples

Starter cultures of 10 ml of LB medium supplemented with carbenicillin (50 μg/ml) and kanamycin (25 μg/ml) were inoculated with *E. coli* M15 (pREP4, pQE30-wtHOD) and grown overnight at 37 °C. The overnight culture was diluted 1:1000 in LB medium supplemented with the above antibiotics and grown at 37 °C until *A*_600_ = 0.6 was reached. After cooling to 24 °C, expression was induced with 0.5 mm isopropyl 1-thio-β-d-galactopyranoside. The culture was then left to shake for a further 20 h, and then cells were collected by centrifugation. Cells were resuspended in 50 mm Tris buffer, pH 7.5, 200 mm NaCl, 20 mm imidazole (Binding Buffer) containing DNase, lysozyme, and protease inhibitor (Roche Applied Science). The cell suspension was sonicated and centrifuged. The supernatant was filtered through a 0.22-μm filter and loaded onto a Ni-FF or HisPrep 16/10 column previously equilibrated with Binding Buffer. The column was washed with Binding Buffer until the absorbance at 280 nm (A_280_) returned to the baseline value. Protein was eluted by applying a gradient of 50 mm Tris buffer, pH 7.5, 200 mm NaCl, 500 mm imidazole. Fractions containing pure HOD were pooled, concentrated, and dialyzed for 16 h against 50 mm Tris buffer, pH 7.5, containing 5 mm NaCl and 2 mm EDTA. The concentrations of WT HOD and its variants were determined spectrophotometrically using an extinction coefficient ϵ_280_ = 64,525 m^−1^ cm^−1^, deduced from its amino acid sequence (23).

##### QND Analyses

QND substrate was synthesized by Sigma (purity 99.6%). For QND deprotonation studies, the substrate was dissolved in DMSO at 150 mm and diluted in 1 mm potassium phosphate buffer, pH 7.6, to a final concentration of 20 μm. The pH was monitored as it was gradually increased by the stepwise addition of NaOH, and absorption spectra (from 300 to 500 nm) of the sample were recorded after each addition. A single p*K_a_* value associated with the spectral change was calculated by fitting the absorbance (*y*) values at 335 and 380 nm to Equations 1 and 2, respectively,

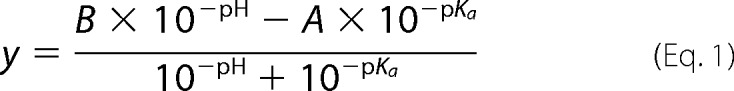


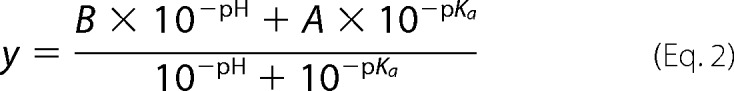
 where *A* and *B* are the highest and lowest absorbance values, respectively.

QND extinction coefficients at 335 nm were determined at different pH values in 0.1 m MTE (MES/Tris/ethanolamine) buffer as follows: 9990 m^−1^·cm^−1^, pH 6; 9920 m^−1^·cm^−1^, pH 6.5; 9910 m^−1^·cm^−1^, pH 7; 9880 m^−1^·cm^−1^, pH 7.5; 9850 m^−1^·cm^−1^, pH 8; 9800 m^−1^·cm^−1^, pH 8.5; 9690 m^−1^·cm^−1^, pH 9; 9320 m^−1^·cm^−1^, pH 9.5; 8220 m^−1^·cm^−1^, pH 10; 6870 m^−1^·cm^−1^, pH 10.5; and 5720 m^−1^·cm^−1^, pH 11.

##### Steady-state Studies

Initial rates of QND substrate oxygenation by HOD were measured by following the absorbance decrease at 335 nm (using the above ϵ_335_ values). The effect of pH on the kinetic parameters was determined over a pH range from 6 to 11 by a series of enzyme activity assays at varying QND concentrations (from 5 to 200 μm) in air-saturated 0.1 m MTE buffer containing 5 mm NaCl and 2 mm EDTA at 20 °C. To prevent any pH shift, reactions were initiated by adding a small amount (5–10 μl) of concentrated HOD samples to reach the desired concentrations 15 nm (WT HOD), 1.6-0.033 μm (D126A), and 32-1.3 μm (H251A). Steady-state kinetic constants were obtained by fitting the initial rates at different QND concentrations to the Michaelis-Menten equation.

The effect of imidazole on WT HOD and its H251A variant was determined in the pH range 6–9 by measuring the enzyme activity with 150 μm QND in the presence of varying concentrations of imidazole (range from 0 to 250 mm) in air-saturated 0.1 m MTE buffer at 20 °C. p*K_a_* values were obtained by fitting the activity ratio (activity in presence and absence of imidazole) to Equation 2 (where, in this case, *A* and *B* are the highest and lowest activity ratio, respectively).

##### Transient-state Studies

Kinetic studies of QND deprotonation by HOD were carried out under anoxic conditions using a stopped-flow spectrometer (Applied Photophysics) maintained in an anaerobic glovebox. Reactants were prepared anaerobically inside the glovebox, and the enzyme was made anoxic by passing it through a PD-10 column. QND deprotonation was measured by following the absorbance increase at 370 nm upon mixing enzyme and substrate. The experiments were carried out in 0.1 m MTE, 5 mm NaCl, and 2 mm EDTA at 5 °C with (concentrations after mixing) 20 μm WT HOD (40 μm for HOD variants) and QND from 40 μm to 1 mm (200 μm to 1 mm for HOD variants). The stopped-flow equipment was fitted with a 5-μl cell to measure higher rates accurately by virtue of the shorter dead time (∼0.5 ms). The observed rate constants (*k*_obs_) were obtained by fitting observed traces (6–16 shots) to a single exponential equation. The *k*_obs_ values obtained at different substrate concentrations were fitted to Equation 3,

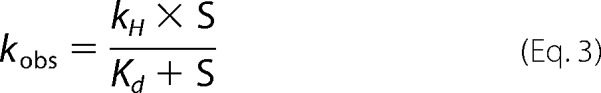
 where *k_H_* is the maximal rate of deprotonation; *K_d_* is the dissociation constant, and S represents the substrate concentration.

To study the effect of pH on QND enzymatic deprotonation, HOD samples were prepared as above, using an MTE buffer at the desired pH. The p*K_a_* values were obtained by fitting the transient-state kinetic constants (*k_H_* or *k_H_*/*K_d_*) obtained at different pH values to Equation 4,

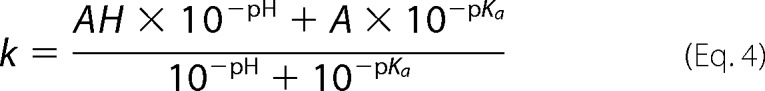
 where *AH* and *A* represent, respectively, the limiting rate constants of the protonated and deprotonated forms of the enzyme-substrate complex (*k_H_ versus* pH plot) and free enzyme and/or free substrate (*k_H_*/*K_d_ versus* pH plot) (24).

QND deprotonation by WT HOD was additionally studied using deuterium oxide (D_2_O) at different pH (pD) values. MTE buffer components were dissolved in D_2_O, and the pD values were adjusted with NaOD or DCl. The former values were determined by adding 0.42 to the pH electrode readings (25). The D_2_O-based buffer was used to prepare the reactants. Protein samples were buffer exchanged using a PD5 column as described above.

##### Crystallographic Studies and in Crystallo UV-visible Spectroscopy

Non-merohedrally twinned (twin law −*h*, *l*, *k*) crystals of HOD H251A belonging to space group *P*2_1_2_1_2_1_ were obtained by the vapor diffusion method as described before (26). Briefly, equal volumes of protein solution (150 mg/ml in 20 mm Tris, 0.1 m NaCl, 2 mm EDTA, 1 mm DTT, pH 7.5) and a reservoir solution containing 1.65 m sodium/potassium tartrate and 0.1 m Hepes, pH 7.0 were mixed at 18 °C. The HOD H251A·QND complex was obtained by soaking crystals for about 2 h in a reservoir solution enriched by a 2 mm solution of the natural substrate QND. Cryoprotection was achieved by quickly passing crystals through a drop of pure perfluorodecalin. A data set was collected at 1.95 Å resolution at the beamline I04 of Diamond Light Source (Didcot). Data processing was carried out using the xia2 (27) pipeline employing XDS (28) and AIMLESS (29) packages. The programs COOT (30) and REFMAC5 (31) were used for model rebuilding and refinement, respectively. Coordinates (code 4CFS) and structure factors have been deposited with the Protein Data Bank. In *crystallo* UV-visible measurements were carried out at 100 K on vitrified crystals using the microspectrophotometer installed at the I29S Cryobench station of the European Synchrotron Radiation Facility (Grenoble, France).

##### Computational Studies

The calculations reported here were done using density functional theory (DFT) methods as implemented in the Gaussian09 program package (32). The unrestricted hybrid DFT method UB3LYP in combination with 6–31g and 6–31g(3d,p) basis sets were used, designated as BS1 and BS2, respectively. The QND deprotonation reaction was studied using models of various molecular sizes. The smallest chemical system contained the QND molecule only and is a mimic for the free QND deprotonation in solution. Based on the coordinates of the anaerobic HOD·QND complex (Protein Data Bank entry 2WJ4), we created active site models of different size, whereby His-251, Asp-126, and the Trp-36 backbone were successively introduced to the system. The largest system studied here constitutes the active site residues and water molecules surrounding the QND substrate as follows: His-251, Asp-126, and Trp-36 backbone, His-38, His-100, Ser-101, and His-102. We also calculated a model for the D126A and H251A variants, whereby Asp-126 was absent for both, and His-251 was removed in the H251A system.

Full geometry optimizations (without constraints for QND molecule) were performed followed by a frequency calculation for all structures. All energies reported in this work were obtained with both basis sets. Free energies were calculated at 1 atm pressure and 298 K temperature and contain entropic and thermal corrections. Transition state searches were started by initially running extensive geometry scans, whereby one degree of freedom for the reaction coordinate (*i.e.* the QND hydroxyl-His-251Nϵ distance for WT and D126A; His-100 for H251A) was fixed and all other degrees of freedom were fully optimized. The maxima of these scans were then used as starting points for the full transition state searches. Solvent calculations were done using the polarized continuum model with either chlorobenzene (dielectric constant ϵ = 5.70) or water (ϵ = 78.35). Previous studies showed that these methods reproduce experimentally determined rate constants of oxygen atom transfer reactions to within 3 kcal·mol^−1^ (33, 34).

To determine the electronic changes in the reaction mechanism and the effect of hydrogen-bonding donors on the proton transfer processes, we evaluated the ionization potential (IE) and the electron affinity of the substrate (EA). In addition, we estimated the acidity of the individual proton acceptor groups as follows. We first calculated the energy to abstract a hydrogen atom to these sites or bond dissociation energy of the O–H/N–H bonds that are broken in the process, BDE_H_. Then we estimated the acidity of the proton acceptor groups of the substrate from the hydrogen atom abstraction energy, the ionization potential of a hydrogen atom (IE_H_), and the electron affinity of the substrate as explained in supplemental Scheme S1, *B* and *C*.

##### QM/MM Calculations

QM/MM calculations were done using previously described methods (35, 36), which are briefly summarize here. Starting from the relevant WT HOD and H251A variant crystal structures (Protein Data Bank entries 2WJ4 and 4CFS, respectively), hydrogen atoms were added to the structure using PDB2PQR program package (37). The D126A mutation was generated *in silico*. We made sure that all arginine and lysine residues were protonated and all glutamic and aspartic acid side chains were deprotonated. Active site histidines were singly protonated. Solvent (with a 35-Å sphere) was added to these structures, and the complete structures were then equilibrated and energy-minimized by a molecular dynamics simulation and heating procedure to 298 K using the CHARMM force field (38). We selected several snapshots from this molecular dynamics simulation as starting points for later QM/MM studies at different time intervals. QM/MM calculations used the widely reported technique implemented in the ChemShell software package of running the CHARMM force field through DL-POLY (39). The QM regions, containing the QND substrate together with the residues listed above for the DFT calculations, were described by TURBOMOLE (40) using the UB3LYP hybrid DFT method in combination with a SV(P) basis set.

## RESULTS

Experimental and computational studies were combined to unravel the first catalytic step of QND oxygenation in the HOD active site, which is proposed to involve the deprotonation of the substrate. Initially, the spectral properties of the isolated QND substrate were measured at different pH values to determine the p*K_a_* value and to discriminate between the different reaction steps. The role of two active site residues, His-251 and Asp-126, was then investigated experimentally by combining site-directed mutagenesis with steady- and transient-state studies, together with the analysis of the HOD H251A·QND complex crystal structure. Finally, energy values for the HOD reaction were obtained by DFT methods to determine the involvement of the above residues in HOD catalysis and the existence of possible transient-state intermediates.

### 

#### 

##### QND Spectral Properties

**FIGURE 1.**
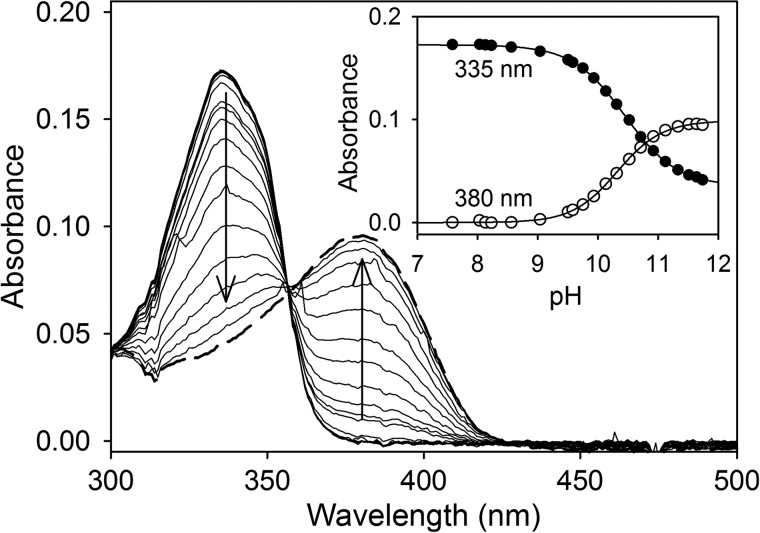
**QND pH titration.** *Main panel*, QND (20 μm) spectral changes during stepwise addition of NaOH in 1 mm potassium phosphate buffer at 25 °C. Spectra at pH 7.6 (*thick line*) and pH 11.5 (*dashed line*). The inset show the data fitting at 335 and 380 nm to Equations 1 and 2, respectively.

To characterize the first step of the postulated reaction mechanism, QND spectral properties were studied to explore the existence of distinguishable UV-visible species during the HOD reaction. To determine the p*K_a_* value of the QND hydroxyl group, the substrate was diluted in 1 mm potassium phosphate buffer, pH 7.6, and the pH was gradually increased by stepwise addition of NaOH, and the UV-visible spectra of the sample were recorded. At pH 7.6, the spectrum of the substrate exhibits one distinctive maximum at 335 nm with a slight shoulder near 350 nm. An increase in the pH results in a bathochromic shift of this peak to 380 nm (Fig. 1). The absorbance values at 335 and 380 nm were plotted against their respective pH values, and by using global fitting, a p*K_a_* value of 10.4 ± 0.1 was calculated (Fig. 1, *inset*). In contrast, when the substrate was mixed aerobically with a small amount of WT HOD (15 nm), using a stopped-flow instrument, the above spectral shift disappeared, although an absorbance decrease at 335 nm was observed (data not shown). This spectral shift corresponds to substrate oxygenation by WT HOD, and under these conditions, the QND deprotonation could not be observed.

##### Steady-state Studies

**FIGURE 2.**
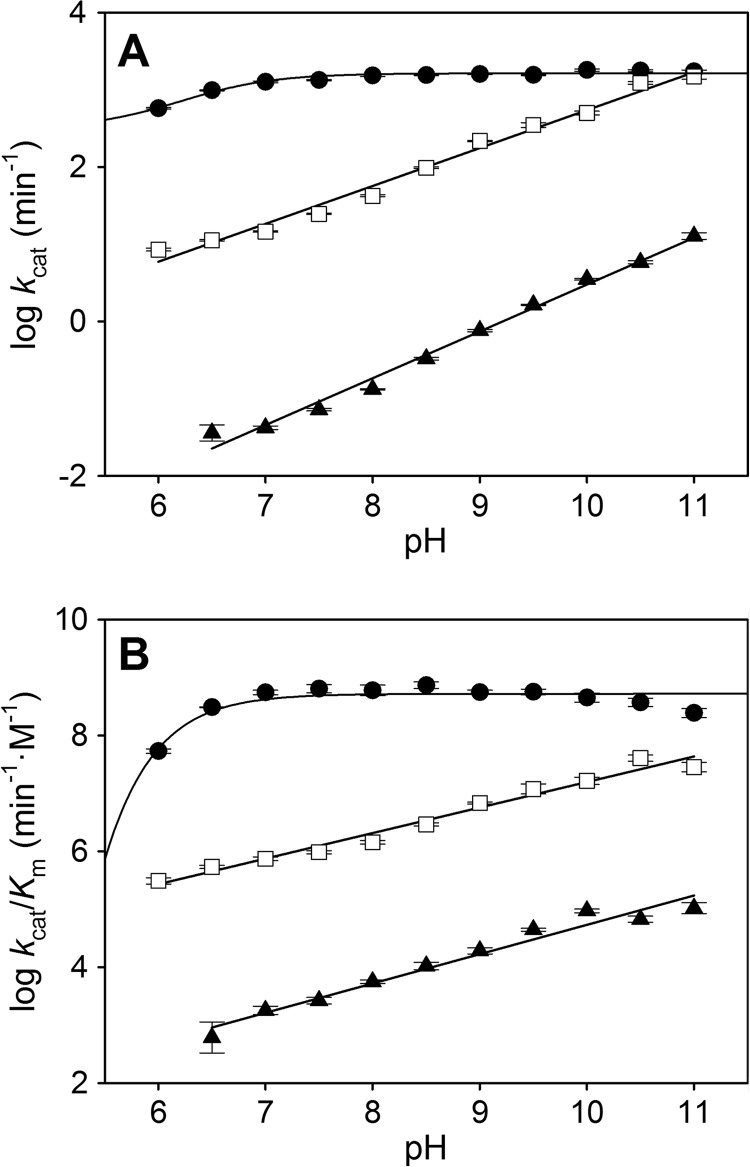
**pH dependence of steady-state kinetic constants for QND oxygenation by WT HOD and its D126A and H251A variants.** pH dependence of *k*_cat_ (*A*) and *k*_cat_/*K_m_* (*B*) for WT (●), D126A (□), and H251A (▴). All reactions were performed in air-saturated 0.1 m MTE buffer, 5 mm NaCl and 2 mm EDTA at 20 °C. The errors considered in the measured parameters were taken larger than the standard deviation between four replicates and the numerical errors after fitting.

Fig. 2 shows the pH dependence of the steady-state kinetic constants for QND oxygenation by WT HOD and its D126A and H251A variants. WT HOD *k*_cat_ (Fig. 2*A*) and *k*_cat_/*K_m_* values (Fig. 2*B*) are nearly constant from pH 7 to 11, showing a slight decrease below pH 7. However, the kinetic constants for both HOD variants increased linearly, in a logarithmic scale, with the pH (Fig. 2, *A* and *B*). Only a single p*K_a_* value of 6.2 ± 0.2 for the WT HOD *k*_cat_ profile could be obtained.

Table 1 compiles the steady-state kinetic constants for WT HOD and its variants at pH 6.5 and 10.5, fully consistent with those previously reported at pH 8 (17). At low pH, the *k*_cat_/*K_m_* values for the D126A and H251A variants are 570- and 512,000-fold lower than for WT HOD. When the pH was increased, the variants remained less efficient than WT HOD, although the difference in *k*_cat_/*K_m_* is 2 orders of magnitude lower (9-fold for D126A and 5500-fold for H251A). Although the *K_m_* values for the D126A variant are similar at different pH values, the *k*_cat_ values increased from 0.19 ± 0.01 s^−1^ (16.3 ± 0.1 s^−1^ for WT) at low pH to 20.5 ± 0.3 s^−1^ (29.5 ± 0.4 s^−1^ for WT) at high pH, showing that D126A variant activity could be partially restored at high pH values. However, the *k*_cat_ for the H251A variant remains 300-fold lower than WT HOD after increasing the pH.

**TABLE 1:**
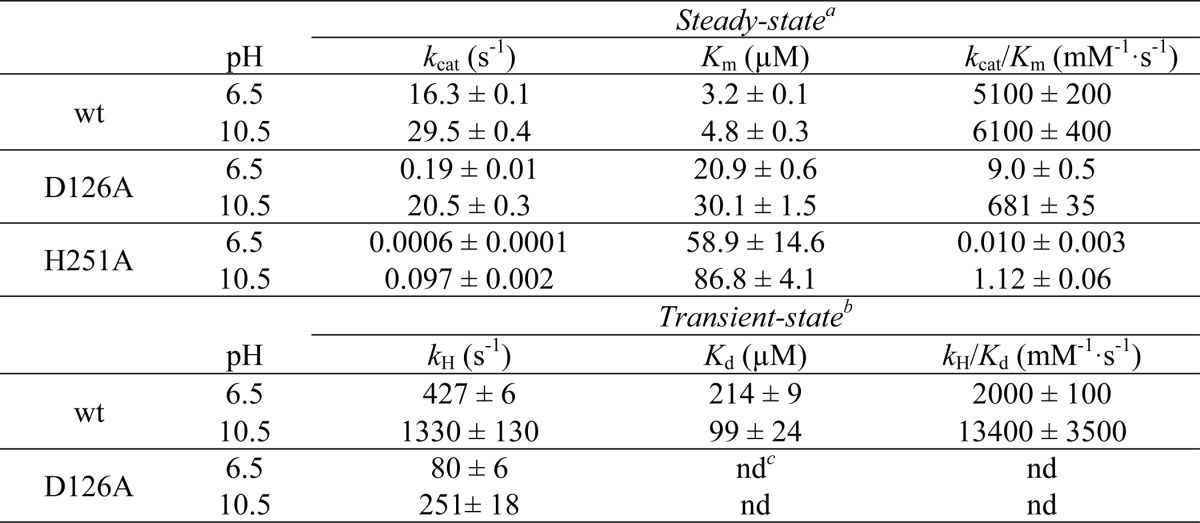
**Steady- and transient-state kinetic constants for WT HOD and its variants at different pH values**

*^a^* Steady-state constants were determined in 0.1 m MTE Buffer containing 2 mm EDTA, 5 mm NaCl at 20 °C.

*^b^* Transient-state constants were determined in the former buffer at 5 °C.

*^c^* nd means not determined because of no QND dependence; values are shown at 1 mm QND. Means ± S.D. from curve fitting are provided.

**FIGURE 3.**
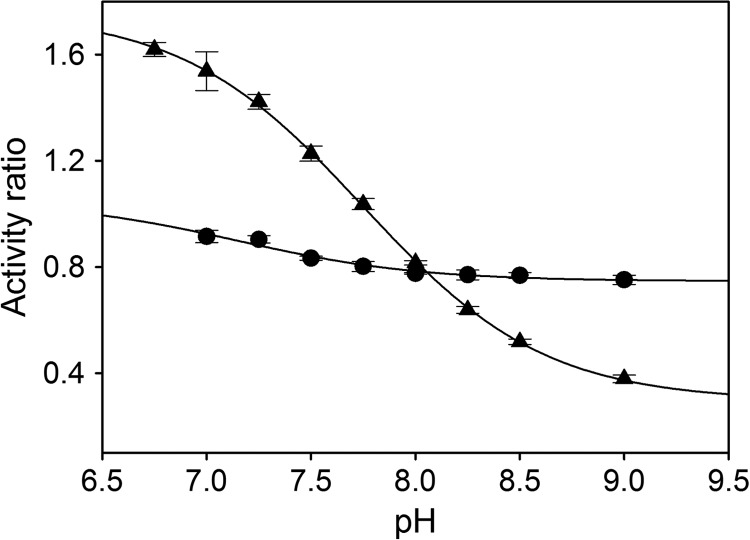
**pH dependence of activity ratio in presence of imidazole.** Ratio calculated from *k*_cat_ determined under saturating imidazole *versus* non-imidazole concentration for WT (●) and H251A variant (▴) in 0.1 m MTE buffer, 5 mm NaCl, and 2 mm EDTA at 20 °C. Data were fit to Equation 2.

The histidine to alanine exchange in the HOD active site is equivalent to removing the imidazole moiety, and for that reason it might be expected that the kinetic properties for the H251A variant would be partially restored in the presence of imidazole. WT HOD activity after imidazole addition decreased in the pH range assayed (Fig. 3), and this inhibition was imidazole concentration-dependent (from 0 to 0.25 m imidazole). For WT HOD, the activity ratio (saturating *versus* zero imidazole concentration) values across the pH range showed a p*K_a_* value of 7.2 ± 0.2. In contrast, H251A activity increased slightly below pH 8 showing a p*K_a_* value of 7.7 ± 0.1. As the protonated form of imidazole only partially restores the activity in the H251A variant and inhibits the activity of both samples at certain pH values, it clearly suggests that the exogenous imidazole is not responsible for QND deprotonation. The inhibition is probably because imidazole is affecting the active site microenvironment, perhaps blocking substrate access for WT HOD and preventing water or other residues to act as possible bases.

##### Transient-state Studies

To provide a more detailed characterization of the QND deprotonation step in the HOD active site, rapid stopped-flow measurements under anoxic conditions were performed, and the pH dependence of the deprotonation rate constant (*k_H_*) and *k_H_*/*K_d_* was investigated for WT HOD and its D126A and H251A variants.

First, by using a photodiode array detector to obtain time-dependent absorbance spectra, we determined the QND spectral shifts that take place after mixing anaerobically QND substrate with WT HOD and its variants (data not shown). As observed previously for QND deprotonation in solution during the pH titration with NaOH (Fig. 1), an absorbance increase at ∼370–380 nm and a decrease at 335 nm was observed for WT HOD and D126A samples. However, for the H251A variant, only a catalytically irrelevant absorbance increase at 315 nm was observed after a 15-min reaction. The H251A variant activity was too low to obtain any meaningful stopped-flow data.

**FIGURE 4.**
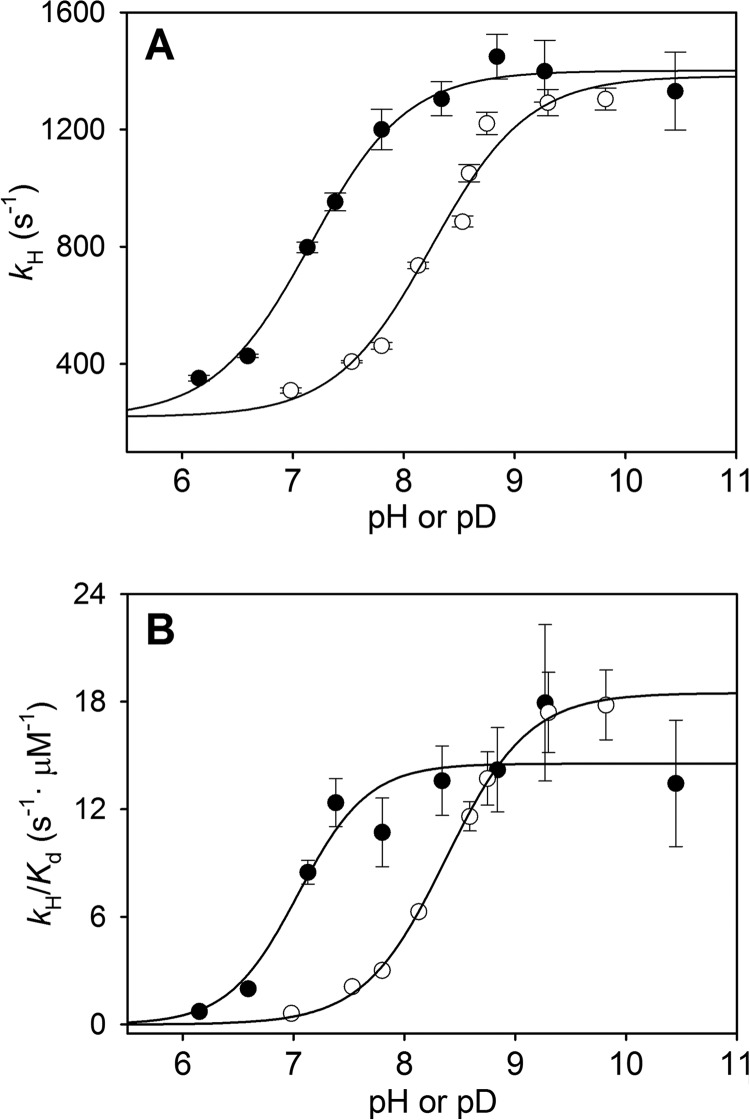
**pH/pD dependence of transient-state kinetic constants for QND deprotonation by WT HOD.** pH/pD dependence of *k_H_* (*A*) and *k_H_*/*K_d_* (*B*) for WT HOD determined anaerobically in presence of water (●) and deuterium oxide (○) in 0.1 MTE buffer, 5 mm NaCl, and 2 mm EDTA at 5 °C. Data were fit to Equation 4.

Transient-state constants (*k_H_*, *K_d_*, and *k_H_*/*K_d_* values) were determined over a pH range from 6.5 to 10.5 for WT HOD (Fig. 4) and the D126A variant (data not shown). For WT HOD, a p*K_a_* value of 7.2 ± 0.1 and 6.9 ± 0.3 was obtained for the *k_H_* and *k_H_*/*K_d_* profiles, respectively. For the D126A variant, no p*K_a_* value could be obtained from its *k_H_* pH profile, as the deprotonation rate remains constant across the investigated pH range, exhibiting only a slight rate increase above pH 9.5. Because of the low solubility of the substrate (soluble only up to a maximum of 1 mm in the stopped-flow reaction cell) and the similar deprotonation rate along the QND concentration range assayed, we were not able to determine any *K_d_* value for this variant.

Table 1 summarizes the transient-state constants for WT HOD and D126A variant at pH 6.5 and 10.5. The comparison between *k*_cat_ (at 20 °C) and *k_H_* (at 5 °C) for both samples reveals that QND deprotonation is not rate-limiting during catalysis, because *k_H_* values were at low and high pH, respectively, 27 and 46 (WT HOD) and 420 and 12 (D126A) times higher than the *k*_cat_ value. Because substrate deprotonation is not rate-limited by other catalytic steps, maximum deprotonation rates under transient-state conditions are attained at higher QND concentrations, causing a *K_d_* value increase with respect to *K_m_* (1 order of magnitude) for WT HOD. The *k_H_* for the D126A variant remains 40–5-fold lower than WT HOD across the pH range assayed (data not shown), in contrast to the partial activity recovery obtained under steady-state conditions (Fig. 2), in agreement with a nonlimiting proton transfer during enzyme turnover.

The above p*K_a_* values for WT HOD concur with the presence of a histidine residue at the active site that acts as catalytic base. However, the p*K_a_* value obtained here was different in comparison with that obtained under steady-state conditions (p*K_a_* 6.2), suggesting again that a different step of the catalytic cycle is limiting the overall turnover rate constant.

Anaerobic QND deprotonation by WT HOD was additionally studied under transient-state conditions using D_2_O at different pD values. The pH and pD dependence for the transient-state kinetic constants is summarized in Fig. 4. The proton transfer is affected by D_2_O in the 7 to 8.5 pH range and an apparent solvent isotope effect of ∼2.25 could be observed, whereas at high pH/pD, the *k_H_* values are similar (Fig. 4*A*). In contrast, QND binding is slightly improved in D_2_O as shown by larger *k_H_*/*K_d_* values at high pD values (Fig. 4*B*). The involvement of a proton transfer is clearly shown by the shift in the p*K_a_* values of 8.3 ± 0.1 for *k_H_* in the D_2_O profile (7.2 ± 0.1 in H_2_O) and 8.4 ± 0.1 for *k_H_*/*K_d_* D_2_O profile (6.9 ± 0.3 in H_2_O), suggesting a significant effect of D_2_O on both the HOD·QND complex (*k*_H_ profile) and the free HOD (*k*_H_/*K_d_* profile) ionization state.

##### Crystallographic Analysis

**FIGURE 5.**
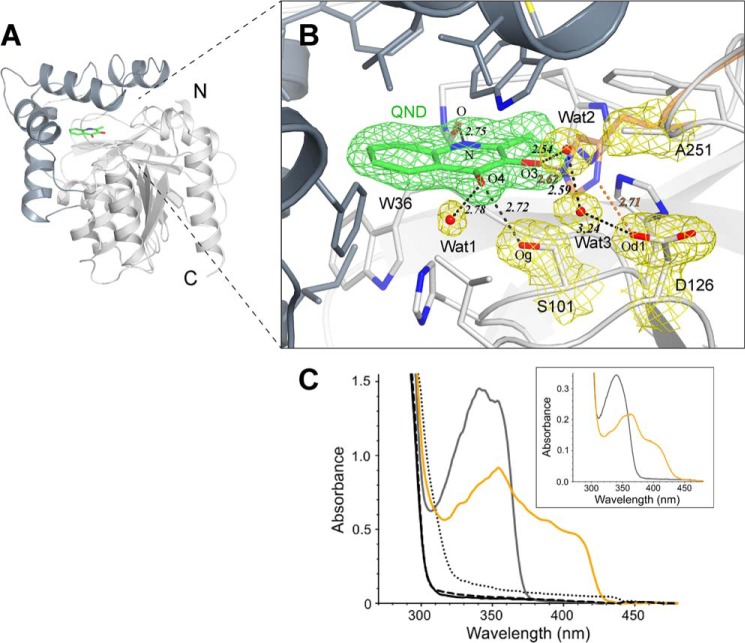
**HOD H251A·QND complex.** *A,* schematic representation of HOD showing the α/β-hydrolase fold core and cap domains (*light* and *dark gray*, respectively). QND substrate bound at active site cavity (*green*). *B,* HOD H251A·QND active site. Residue side chains (*gray sticks*), water molecules (*red spheres*), and hydrogen bonds (*black dashed lines*), together with the 2m*F_o_* − D*F_c_* electron density maps (1.0σ level) for QND molecule (*green*) and for water molecules, Ser-101, Asp-126, and Ala-251 residues (*yellow*), are shown. For the WT HOD·QND complex, His-251 side chain (*orange sticks*) and distances (in Å, as *orange dashed lines*) are shown. *C, in crystallo* UV-visible spectra of WT HOD (*black*), H251A (*dashed*), HOD·*N*-acetyl-anthranilate product complex (*dotted*), HOD H251A·QND complex (*gray line*), and anaerobic WT HOD·QND complex (*orange line*) at pH 7 and 100 K. The *inset* shows the spectra in solution for anaerobic HOD H251A·QND (*gray line*) and WT HOD·QND (*orange line*) complexes at pH 8.

To provide a firm structural basis to the observation that the H251A replacement essentially abolishes catalytic activity, we solved the x-ray structure of this variant in complex with the QND substrate at 1.95 Å resolution. Data processing and refinement statistics are given in Table 2. Overall, the HOD H251A·QND complex is essentially identical to the WT HOD·QND complex solved previously under anaerobic conditions (17). QND binds in the catalytic pocket buried at the interface between the α/β-hydrolase fold core domain and the cap domain (Fig. 5*A*). The binding mode is unaffected by the H251A replacement (Fig. 5*B*). QND is held in position by a combination of hydrogen bonds and hydrophobic interactions. QND carbonyl oxygen (O4) is stabilized by a hydrogen bond (2.72 Å) with the triad's nucleophile Ser-101, whereas its NH group bonds Trp-36 carbonyl oxygen (2.75 Å). The only significant consequence of the H251A substitution is the disruption of the hydrogen bonding network connecting the QND OH group to the His-251/Asp-126 dyad. Two solvent molecules (*Wat2* and *Wat3* in Fig. 5*B*) loosely occupy the position of His-251 side chain thereby engendering an alternative network connecting QND OH group to the carboxylate group of Asp-126. However, the lack of activity for the H251A variant suggests that this water-mediated network is unable to sustain QND deprotonation. To compare crystallographic observations with solution properties, we carried out *in crystallo* micro-spectrophotometric UV-visible measurements at 100 K and pH 7.0. As shown in Fig. 5*C,* the HOD H251A·QND complex spectrum displays a peak at 341 nm with a shoulder at 354 nm. The spectrum of the complex recorded in solution is essentially identical (Fig. 5*C, inset*). However, the *in crystallo* spectrum of the anaerobic WT HOD·QND complex exhibits a substantial bathochromic shift with broad features extending to 420 nm and a concomitant decrease of the 341 nm band (similar spectrum is observed in solution). This is consistent with deprotonation of QND OH group by WT HOD His-251. Spectra of control WT HOD, H251A variant and WT HOD·product are all essentially featureless above 300 nm (Fig. 5*C*). Taken together these results indicate that in the crystal as well as in solution, His-251 is responsible for the deprotonation of QND OH group.

**TABLE 2:** **HOD H251A·QND complex data collection and refinement statistics**

Data collection	
Beamline	I04 (Diamond Light Source)
Wavelength	0.9763
Resolution range/Highest resolution bin	46.45 to 1.95 Å/(2.00 to 1.95 Å)
Space group	*P*2_1_2_1_2_1_
Cell dimensions	44.73, 166.85, 167.74 Å
Unique reflections	92,811
Overall redundancy	7.73/(7.84)
Completeness	100.0%/(100.0%)
Wilson B	24.47 Å^2^
*R*_symm_	8.6%/(6.3%)
〈*I*/σ(*I*)〉	14.5/(2.9)

**Refinement**	
Protein Data Bank code	4CFS
*R*_factor_/*R*_free_	16.3%/19.8%
Twinning law/fraction	−*h,l,k*/0.489
No. of non-H atoms	9772
Mean *B* value	29.94 Å^2^
Root mean square deviation bond lengths	0.008 Å
MolProbity score	1.44 (97% percentile)

##### Theoretical Calculations

DFT and QM/MM methods were employed to gain insight into the role of key active site residues. Our smallest chemical system, free QND, mimics the deprotonation reaction in solution. Subsequently, the model was expanded to include active site residues His-251, Asp-126, and the Trp-36 backbone. The latter stabilizes QND in the active site by H-bonding the NH of the heterocycle (Fig. 5*B*). Our largest chemical models mimic the active sites of WT HOD and its D126A and H251A variants.

##### Free Substrate DFT Calculations

To characterize the QND spectral shifts that were observed during the pH titration in solution (Fig. 1), free QND deprotonation and consequently the acidity of the substrate was investigated using two different strategies (supplemental Scheme S1). First, the deprotonation reaction from both NH and OH substrate groups was investigated in water (supplemental Scheme S1*A*), as well as the double deprotonation mechanism. Second, the electrophilicity of the QND groups was studied with respect to hydrogen atom abstraction. This was performed by calculating the bond dissociation energy of the O–H/N–H bond (BDE_H_), the electron affinity of the substrate (EA), and the acidity (Δ*G*_acid_).

Energy values obtained with the first strategy suggest that QND-NH deprotonation is not a spontaneous process. However, it is more favorable than OH deprotonation, showing an ∼6 kcal·mol^−1^ (B3LYP/BS2) energy difference (0.25 kcal·mol^−1^ in solvent). A similar trend is observed for the second deprotonation reaction.

When the second strategy was applied, the values obtained (supplemental Table S1) show similar behavior, with an energetic preference for QND-NH deprotonation over QND-OH deprotonation. In this case, the calculated acidity (Δ*G*_acid_) for the NH groups is ∼12 kcal·mol^−1^ lower in energy than that found for the OH group. The second deprotonation reaction is favored after the first deprotonation takes place.

##### DFT Calculations on HOD Small Systems

**FIGURE 6.**
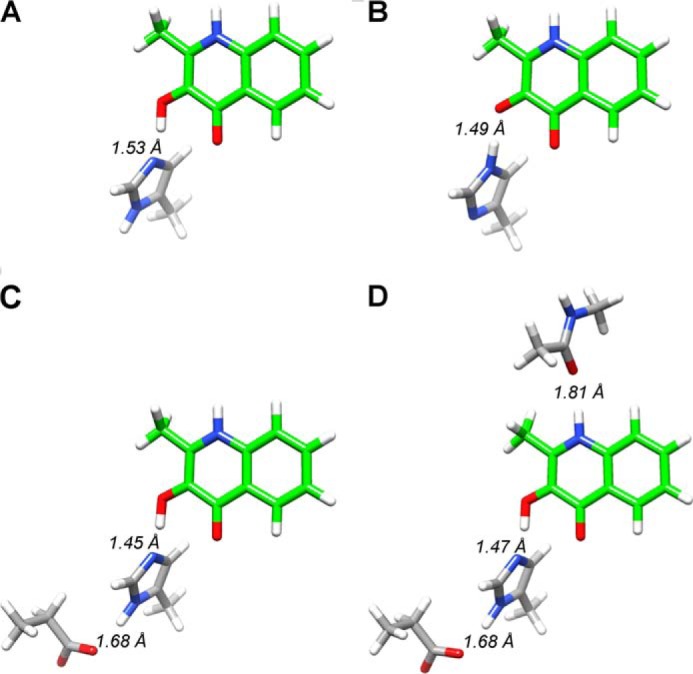
**DFT systems.** The DFT systems studied include QND molecule (*green*) and protonated or deprotonated His-251 (*A* and *B*), Asp-126 (*C*), and Trp-36 backbone (*D*).

Using the coordinates from the HOD·QND complex crystal structure (Protein Data Bank entry 2WJ4), we studied the involvement of different HOD active site residues on substrate deprotonation. Four different small systems were considered, where Asp-126 and the Trp-36 backbone were successively added (Fig. 6).

The values listed in Table 3 were obtained from calculations of the energy differences between the protonated and deprotonated QND hydroxyl group by His-251(Nϵ). Electrostatic interactions several angstroms away from the proton transfer site seem to have major thermodynamic effects on the reaction, with a double-unprotonated His-251 abstracting the hydroxyl proton the easiest (largest exergonic reaction) and the single protonated His-251 requiring a considerable energy for proton abstraction. For QND substrate with a nearby proton acceptor group, we find the following trend in proton abstraction ability: His-251^−^ > His-251–Asp-126 > His-251–Asp-126–Trp-36 > His-251(H). Overall, these calculations suggest that the His-251(Nδ) protonation state is unfavorable and probably decreases the basicity of the residue. However, when Asp-126 is present, the exothermicity of the proton transfer reaction increases (∼11 kcal·mol^−1^) with the Trp-36(CO)–QND(NH) hydrogen bond having little effect (∼2 kcal·mol^−1^). The most basic structure and consequently the best base for the reaction is a fully deprotonated His-251.

**TABLE 3:** **QND deprotonation energies for different HOD systems**

	Δ*E* + ZPE	Δ*G*
His-251^−^	−10.1 (−5.7)	−10.0 (−5.7)
His-251(H)	13.4 (7.5)	13.3 (7.4)
His-251–Asp-126	1.8 (3.6)	1.8 (3.6)
His-251–Asp-126–Trp-36	3.6 (4.5)	3.6 (4.6)
WT	−0.9 (−4.0)	−0.8 (−3.9)
D126A	13.1 (−5.4)	14.0 (−4.5)
H251A	28.0 (14.8)	27.8 (14.6)

Zero point (ZPE) and Gibbs free energies for QND deprotonation reaction using UB3LYP and 6–31g(3d,p) basis set. Solvent corrected values are shown in parentheses. Energies are in kcal·mol^−1^.

We also calculated the BDE_H_, EA, and Δ*G*_acid_ energies (supplemental Table S1) for these systems, which generally confirm the trends of the above calculations. In this case, the Δ*G*_acid_ differences in solvent between His-251(H) and His-251–Asp-126 is ∼12 kcal·mol^−1^, whereas it is ∼4 kcal·mol^−1^ for His-251–Asp-126-Trp-36 *versus* His-251–Asp-126. Therefore, a hydrogen-bonding aspartic acid group toward a histidine increases its proton affinity by about 12 kcal·mol^−1^. We analyzed the physicochemical variables that contribute to the Δ*G*_acid_ change and found that these contain a major component for the electron transfer (∼60 kcal·mol^−1^) and a smaller component for the proton transfer (∼20 kcal·mol^−1^). The electron withdrawing ability of the aspartic acid group therefore pulls extra positive charge in its direction and favors a protonated histidine.

##### DFT Calculations for WT HOD and Its Variants

**FIGURE 7.**
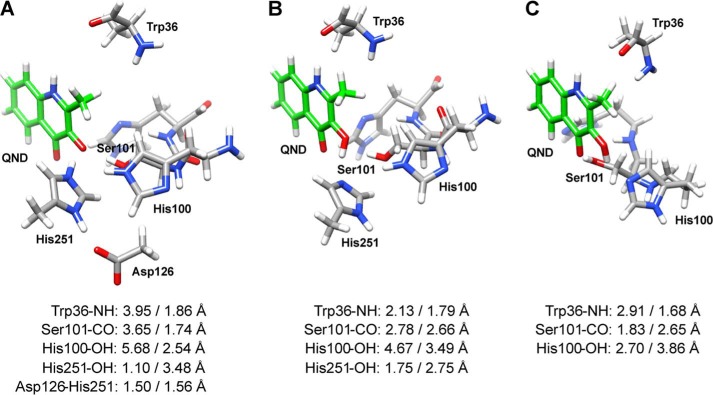
**DFT optimized geometries for WT HOD and its variants.** WT HOD (*A*), D126A (*B*), and H251A (*C*) systems were optimized using B3LYP and BS1 basis set. Distances listed (between active site residues and QND atoms) were taken from the above optimizations (*left*) and from QM/MM optimizations (*right*).

The QND deprotonation step for the WT HOD active site was studied using a QM cluster model described above. The optimized geometries for WT HOD, D126A, and H251A systems are shown in Fig. 7. As can be seen, after optimization the OH proton is transferred to His-251 in the WT system (Fig. 7*A*), but it remains on the substrate in the D126A and H251A systems (Fig. 7, *B* and *C*). In all cases, the substrate moves away from its original position (relative to the Trp-36 carbonyl and Ser-101 hydroxyl groups) being closer to His-251 (WT and D126A) or His-100 (H251A). In the H251A system, His-100 is the only residue favorably positioned for proton abstraction from QND.

The calculations show that the reactions are barrier-less and either exothermic, *i.e.* spontaneous, or endothermic throughout. As expected, the WT system gives an exothermic proton transfer reaction of ∼1 kcal·mol^−1^, although both HOD variants give an endothermic reaction by ∼14 and ∼28 kcal·mol^−1^ for D126A and H251A, respectively. A calculation of the Δ*G*_acid_ values (supplemental Table S1) for these species follows the general trend reported in Table 3. Also, the difference in acidity (Δ*G*_acid_) of the histidine residue is mainly as a result of the large changes in the electron affinity of the substrate.

##### QM/MM Calculations

As subtle changes in geometry and the size of the model appear to give major differences in energetic trends, we decided to follow up our studies with a set of detailed QM/MM calculations. After setting up our system (see “Experimental Procedures” for details), we took three snapshots at 300, 400, and 500 ps from the molecular dynamics simulations. The coordinates for the optimized QM/MM structures, with lowest energy values, are listed in the supplemental material. Substrate positioning at the active site is scarcely affected after replacing either His-251 or Asp-126 with an Ala group, because most key hydrogen-bonding interactions within the active site are maintained. The positioning of His-251 seems to be less restricted in the D126A variant, and for that reason the former residue is closer to the QND-OH, in comparison with WT HOD, where the hydroxyl group is turned toward His-100.

Fig. 7 illustrates the large variation between the DFT models and QM/MM optimized structures with key bond lengths shown. A comparison of the WT HOD structures gives hydrogen-bonding interactions between the carbonyl group of Trp-36 with QND-NH and between the hydroxyl group of Ser-101 with QND-O after QM/MM optimization, but these interactions are lost in the DFT calculations. However, the QND-OH positioning relative to His-251 is more favorable for proton abstraction in the DFT model than in the QM/MM structure. Although we added several constraints to the structure of our DFT system, we had to allow sufficient substrate movement to enable reasonable changes for the proton transfer process.

## DISCUSSION

Cofactor-independent dioxygenases are capable of catalyzing the energetically unfavorable incorporation of molecular oxygen into a substrate in the absence of any metal ions or organic cofactors. Like the homologous QDO, HOD belongs to the α/β-hydrolase fold, a superfamily typically involved in the catalysis of ester or peptide hydrolysis reaction. Thus, these enzymes offer a remarkable example of the chemical versatility of this common protein fold. Oxygenation of *N*-heteroaromatic substrates by HOD and QDO has been proposed to involve an initial substrate deprotonation by the His/Asp dyad (17, 21) subset of the catalytic triad typically employed by the α/β-hydrolase fold superfamily for catalysis. In this work, we have used a multidisciplinary approach to provide a detailed characterization of the first catalytic step.

**SCHEME 2.**
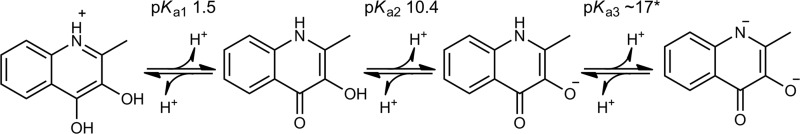
**QND ionization.** Below pH 3, QND substrate is double protonated on *N*1, from pH 3 to pH 9 is neutral, and above pH 11 is single deprotonated. p*K_a_*_1_ value is from Ref. 21; p*K_a_*_2_ value was obtained in this work, and hypothetical (*) p*K_a_*_3_ value in concentrated hydroxide solution is from Ref. 42.

Spectral changes of QND substrate at different pH values were similar to those observed for the enzyme-bound QND under anaerobic *in crystallo* and transient-state conditions, indicating that it is possible to distinguish QND deprotonation from other catalytic steps. The QND substrate possesses two possible ionizable groups, OH and NH, which may be deprotonated during the pH titration with a measured p*K_a_* value of 10.4. Previous studies reported two p*K_a_* values (1.5 and 11) for QND ionization, and these values were assigned to the NH and OH deprotonation, respectively (21). Based on the reasoning below, we suggest a different ionization process, summarized in Scheme 2, in which the observed spectral changes at basic pH values correspond unequivocally to hydroxyl group deprotonation (p*K_a_* 10.4–11), whereas at acid pH, the NH protonation (p*K_a_* 1.5) takes place. Although our computational calculations were not able to accurately predict the preferential OH *versus* NH deprotonation in solution, even employing a methodology used successfully on a different system (41), the p*K_a_* values for the tyrosine hydroxyl group (∼10, similar to QND OH group) and for the indole NH group (∼17, similar to the QND NH group) (42) support the ionization process shown in Scheme 2.

Moreover, the QM/MM optimized structures obtained here together with the HOD crystal structures (17) show that NH deprotonation would not take place during the enzymatic reaction as it is hydrogen bonded to the Trp-36 backbone and points away from any residue that could act as base. Moreover, the QND hydroxyl group is at hydrogen bonding distance to His-251, which may be able to abstract a proton as discussed below.

Highly conserved active site residues among the α/β-hydrolase family form a triad consisting of nucleophilic/histidine/acid residues (18, 19). The nucleophile (Ser-101 and Ser-95 in HOD and QDO, respectively), typically critical for catalysis in α/β-hydrolase fold enzymes as it forms a covalent alkyl-enzyme ester intermediate, has been shown dispensable for the oxygenation reaction discussed here on the basis of mutagenesis work (16, 17). It does, however, contribute to the stabilization of the organic substrate in the active site. Dioxygenation of *N*-heteroaromatics at the α/β-hydrolase fold has been proposed to follow the principles of general base catalysis with the His/Asp subset of the triad playing a critical role in substrate deprotonation/activation for subsequent catalytic steps (17). It has been recently reported that substrate activation is followed by single electron transfer from the deprotonated substrate to oxygen to form a radical pair that recombines to a C2-peroxide intermediate (44).

Our results show that a proton transfer reaction takes place during the first step of the HOD catalytic mechanism, which was observed under anoxic transient-state conditions. The substrate deprotonation is not rate-limiting as suggested by the 15-fold lower *k*_cat_ (20 °C) compared with *k_H_* (5 °C) at low pH for WT HOD. The similar p*K_a_* values (∼7.1) obtained for the HOD·QND complex (*k_H_ versus* pH plot) and the free enzyme (*k_H_*/*K_d_ versus* pH plot) obtained under transient-state conditions suggest the following: (i) the presence of an ionizable residue that needs to be deprotonated in order for catalysis to happen that could be attributed to the His/Asp dyad, as discussed later; and (ii) the p*K_a_* value perturbation for the free QND substrate from 10.4 to a physiological pH range. This shift in p*K_a_* value is analogous to that seen with amine dehydrogenases where the high p*K_a_* value (∼10) of the free substrate is lowered by ∼3 pH units in the enzyme·substrate complex to enable the enzyme to turn over the free base form of the amine at physiological pH values (45). Additional experiments in the presence of D_2_O indicated a p*K_a_* shift of 1.2 units (from ∼7.1 in water to ∼8.3 in deuterium oxide) and an apparent solvent isotope effect of ∼2.25 (in the pH range 7 to 8.5), consistent with a proton transfer reaction. The latter can be attributed to altered force constant effects on the observed p*K_a_* as a result of deuteration. A similar p*K_a_* shift has been reported for trimethylamine dehydrogenase, where substrate ionization was perturbed following deuteration (46). Previous isotope effect studies under steady-state conditions did not reveal a p*K_a_* shift nor any effect on catalytic constants (22). As we have shown here, this is because the proton transfer is not rate-limiting during steady-state turnover and, as such, any isotope effects will be masked under steady-state turnover conditions. The observed kinetic parameters for the H251A and D126A variants clearly confirmed the involvement of the His/Asp dyad in HOD catalysis. The absence of any observable QND deprotonation for the H251A variant during transient-state studies, together with the 6 (low pH) and 4 (high pH) orders of magnitude decrease on the kinetic constants, confirms the role of His-251 as a catalytic base. The x-ray structure of the HOD H251A·QND complex presented here together with the structure of the anaerobic WT HOD·QND elucidated previously (17) highlight that the H251A replacement affects exclusively the hydrogen bonding network connecting the OH group of the substrate to the His/Asp dyad with no alteration on the substrate binding geometry. Transient-state studies clearly show the involvement of Asp-126 during substrate deprotonation (facilitating the His-251 proton abstraction), as shown by the absence of any detectable p*K_a_* value together with a 5-fold lower *k_H_* value at high pH. The steady-state pH profiles for the above variants show an increase of about 0.5 log units per pH unit. If the reaction was simply dependent on the amount of deprotonated QND present in solution, the plots should have unity slopes. Similar trends have been reported for the K9M variant of urate oxidase (47). The absence of any experimentally detectable p*K_a_* value for both His/Asp variants, in the physiological pH range, led us to conclude that the p*K_a_* values for the corresponding enzyme·QND complexes are most likely higher than the p*K_a_* value for the free substrate. In our studies, substrate deprotonation in solution clearly enhances HOD catalysis and partially rescues the D126A variant activity. Previous studies on a H466A variant of choline oxidase showed that activity could be successfully increased at low pH after addition of imidazole (48). However, external addition of imidazole showed only minor or even inhibitory effects on H251A (HOD) activity. This lack of efficient chemical rescue might be attributed to imidazole hindering access of substrate to the active site of HOD.

Computational studies using different DFT active site models, which mimic the active sites of WT HOD, D126A, and H251A variants, have confirmed the experimentally reported reactivity differences. Although the QND substrate position is not maintained when different DFT models are used, *i.e.* Ser-101 and Trp-36, backbone hydrogen bonds are lost in WT HOD and D126A variant, an exothermic proton transfer for WT HOD and an endogenous transfer for both variants could still be observed. The above hydrogen bonds have minor contributions in energetic terms, as shown by the effect of the Trp-36 backbone residue in the small DFT systems, but could be essential for substrate positioning because these hydrogen bonds remained intact after QM/MM optimizations. However, the position of QND after DFT optimization shows that His-251 is the only active site histidine residue that could act as a catalytic base. Furthermore, when the proton was transferred to His-100, as in the H251A system, high energy barriers were obtained. The computational studies allowed us to clarify the role of Asp-126 in HOD catalysis. When Asp-126 was positioned in the proximity of His-251, the proton transfer from QND to His-251 was favored resulting in an ∼15 kcal·mol^−1^ (large systems) and ∼11 kcal·mol^−1^ (small systems) energy reduction. Therefore, a hydrogen-bonding aspartic acid group toward a histidine essentially increases the His-251 proton affinity. In agreement, computational calculations on serine proteases suggested that the triad aspartic residue contributes to catalysis by stabilizing the charges on the conserved histidine residue (49).

We can conclude from the above data that the HOD (and probably QDO taking into account its related structure and previous mutagenesis studies (16, 17, 50)) employs an His/Asp dyad for catalysis. Noteworthy, in C-C hydrolases, a conserved histidine residue acts as a base, facilitating the deprotonation of either a serine or a water molecule, which is then involved in a nucleophilic attack on the substrate molecule (51). A similar role for a histidine residue and catalytic mechanism was postulated for *Hevea brasiliensis* hydroxynitrile lyase based on its crystal structure (52). Structure-function studies on *R. reniformis* luciferase, the only α/β-hydrolase family member that catalyzes an oxygenation reaction, suggested that a conserved histidine residue can act as a catalytic base, but the exact role of the triad residues is not yet fully understood (53).

The initial proton abstraction from the substrate by a catalytic base appears a common step in cofactor-free oxidases/oxygenases and some metal-dependent dioxygenases, which do not share sequence or structure similarities with the above enzymes (13). The crystal structure of the vancomycin biosynthetic enzyme DpgC suggested that an activated water molecule can abstract a proton from the substrate to form an anion intermediate (54, 55). Moreover, the postulated reaction mechanism for copper-dependent quercetin 2,3-dioxygenase starts by proton abstraction from the substrate hydroxyl group to Glu-73 (15, 56). Additionally, non-heme iron-dependent extradiol cathecol dioxygenases contain an active site histidine that could act either as a catalytic base to deprotonate the substrate or by contributing to oxygen activation (57).

**SCHEME 3.**
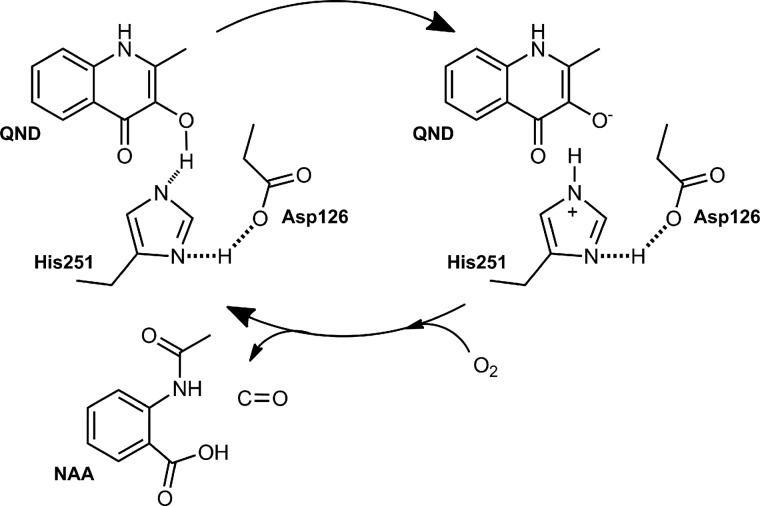
**HOD catalytic cycle.** During the first step of QND oxygenation, the QND-OH proton is abstracted by His-251(Nϵ), with the contribution of Asp-126, which hydrogen bonded the His-251(Nδ). The deprotonated/activated substrate is later attacked by oxygen leading to *N*-acetyl-anthranilate (*NAA*) product formation.

We have used a multidisciplinary approach to characterize the first step during HOD catalysis, which is essential for the subsequent reaction with oxygen. In full agreement with a previous proposal based on structural work (17), we conclude that the His/Asp dyad is crucial for catalysis with His-251 acting as a catalytic base, abstracting a proton from the QND substrate. The proton abstraction is facilitated by Asp-126, which hydrogen bonds to the His-251 residue, thus increasing its basicity (Scheme 3). The study paves the path for further multidisciplinary analyses on cofactor-free dioxygenases, particularly in relation to the steps involving dioxygen. It also illustrates the power of uniting experimental and computational studies in mechanistic enzymology.

## Supplementary Material

Supplemental Data

## Footnotes

5 The abbreviations used are:

HOD: (1*H*)-3-hydroxy-4-oxoquinaldine 2,4-dioxygenase
QDO: (1*H*)-3-hydroxy-4-oxoquinoline 2,4-dioxygenase
QND: (1*H*)-3-hydroxy-4-oxoquinaldine
DFT: density functional theory
QM/MM: quantum mechanics/molecular mechanics
EA: electron affinity
BDE: the bond dissociation energy
QM/MM: quantum mechanics/molecular mechanics.
