# Supplementary material for: Origin of the Proton-transfer Step in the Cofactor-free (1H)-3-Hydroxy-4-oxoquinaldine 2,4-Dioxygenase: EFFECT OF THE BASICITY OF AN ACTIVE SITE HIS RESIDUE
Source: J Biol Chem. 2014 Jan 30;289(12):8620–32. doi: 10.1074/jbc.M113.543033 (PMC3961685; doi:10.1074/jbc.M113.543033)
Supplement: Supplemental Data [file supp_289_12_8620__index.html]

Origin of the proton-transfer step in the cofactor-free 1-H-3-hydroxy-4-oxoquinaldine 2,4- dioxygenase: Effect of the basicity of an active site His residue — Origin of the Proton-transfer Step in the Cofactor-free (1H)-3-Hydroxy-4-oxoquinaldine 2,4-Dioxygenase — Mechanistic Studies on HOD, a Cofactor-free Dioxygenase — Supplemental Data 

# Origin of the Proton-transfer Step in the Cofactor-free (1*H*)-3-Hydroxy-4-oxoquinaldine 2,4-Dioxygenase

## Supplemental Data

**Files in this Data Supplement:**

- Supplemental data (.docx, 566 KB) - Supplemental data
