# Supplementary material for: Origin of the Proton-transfer Step in the Cofactor-free (1H)-3-Hydroxy-4-oxoquinaldine 2,4-Dioxygenase: EFFECT OF THE BASICITY OF AN ACTIVE SITE HIS RESIDUE
Source: J Biol Chem. 2014 Jan 30;289(12):8620–32. doi: 10.1074/jbc.M113.543033 (PMC3961685; doi:10.1074/jbc.M113.543033)
Supplement: Supplemental Data [file supp_M113.543033_jbc.M113.543033-1.docx]

**Supplementary Information for Manuscript:**

Origin of the proton-transfer step in the cofactor-free 1-*H*-3-hydroxy-4-oxoquinaldine 2,4-dioxygenase: Effect of the basicity of an active site His residue

**Aitor Hernandez-Ortega^1^, Matthew G. Quesne^1^, Soi Bui^2^, Dominic P. H. M. Heuts^1^, Roberto A. Steiner^2^, Derren J. Heyes^1^, Sam P. de Visser^1^ and Nigel S. Scrutton^1^**

^1^Manchester Institute of Biotechnology, The University of Manchester, Manchester M1 7DN, United Kingdom

^2^Randall Division of Cell and Molecular Biophysics, King’s College London, London SE1 1UL, United Kingdom

**Contents:**

Supplemental **Table S1**

Supplemental **Scheme S1**

Supplemental Computational Data including: cartesian coordinates for optimized DFT structures (QND substrate and its anions, small systems and wt/D126A/H251A systems) and QM region at 300, 400 and 500 ps for wt HOD, D126A and H251A variants.

| **TABLE S1** | | | | | |  |  |
| --- | --- | --- | --- | --- | --- | --- | --- |
| **EA, BDE_H_ and Δ*G*_acid_ energies for different HOD systems** | | | |  |  |  |  |
| Energies calculated using two different UB3LYP basis sets. Solvent and water corrected values, respectively, between parentheses. Energies in kcal·mol^-1^ | | | | | | |  |
| 6-31g | EA | BDE_H_ | | | Δ*G*_acid_ | | |
| O-H first | -1.2 (35.1, 43,5) | -70.8 (-73.2, -65.3) | | | -242.3 (-197.5, -189.3) | | |
| N-H first |  | -82.7 (-82.2, -82.2) | | | -230.4 (-181.8, -172.4) | | |
| N-H second | -98.9 (4.8, 25.1) | -44.1 (-51.3, -53.4) | | | -171.4 (-181.6, -183.3) | | |
| O-H second | -111.6 (-4.0, 17.6) | -47.8 (-51.0, -51.8) | | | -154.9 (-173.0, -176.9) | | |
| His251^-^ | -86.7 | -114.2 | | | -113.5 | | |
| His251-H | -23.8 | -95.9 | | | -194.6 | | |
| His251-Asp126 | -66.4 | -109.9 | | | -138.1 | | |
| His251-Asp126-Trp36 | -65.8 | -106.9 | | | -141.6 | | |
| wt | -42.8 | -85.1 | | | -186.4 | | |
| D126A | 1.7 | -85.4 | | | -230.7 | | |
| H251A | 5.9 | -82.1 | | | -238.1 | | |
| 6-31g(3d,p) | EA | | BDE_H_ | | Δ*G*_acid_ | | |
| O-H first | -0.6 (36.2*,* 46.7) | -74.9 (-77.1, -69.1) | | | -238.7 (-187.2, -185.7) | | |
| N-H first |  | -79.5 (-79.1, -79.2) | | | -234.1 (-185.1, -175.5) | | |
| N-H second | -96.1 (13.7*,* 27.7) | -44.5 (-44.7, -53.5) | | | -173.7 (-197.1, -185.3) | | |
| O-H second | -108.3 (-1.07*,* 20.6) | -50.6 (-52.8, -53.3) | | | -155.4 (-174.2, -178.4) | | |
| His251^-^ | -81.1 (8.2*,* 26.4) | -112.1 (-108.8, -106.9) | | | -121.2 (328.6, 291.6) | | |
| His251-H | -18.9 (23.1*,* 32.1) | - 96.9 (-93.6, -92.5) | | | -198.5 (298.5, 271.5) | | |
| His251-Asp126 | -58.2 (15. 9*,* 30.8) | -108.1 (-98.9, 95.9) | | | -148.1 (311.1, 276.2) | | |
| His251-Asp126-Trp36 | -59.7 (10.1*,* 26.4) | -105.9 (-96.7, -94.1) | | | -148.7 (314.8, 278.8) | | |
| wt | -38.7 (-34.9) | -79.6 (-57.7) | | | -196.1 (320.8) | | |
| D126A | 6.2 (9.2) | -72.7 (38.7) | | | -247.8 (257.7) | | |
| H251A | 7.6 (8.2) | -82.4 (-60.2) | | | -239.6 (280.1) | | |





**Scheme S1.** QND deprotonation studies and energies definitions. *Top*, Scheme of QND deprotonation on NH or OH bonds. *Bottom*, definitions of BDE_OH_, EA, IE_H_ and Δ*G*_acid_ energies for both protons.

Cartesian coordinates (in Å) for **QND substrate**:

6 3.193817000 -0.835042000 0.004775000

6 2.011343000 -1.546237000 0.006564000

6 0.781680000 -0.858212000 0.004602000

7 -0.411750000 -1.540960000 0.006479000

6 -1.646987000 -0.922572000 0.004868000

6 -1.701760000 0.439066000 0.001073000

6 -0.507272000 1.268769000 -0.001439000

6 0.766199000 0.554452000 0.000748000

6 1.988570000 1.251528000 -0.000933000

6 3.189066000 0.572594000 0.001016000

6 -2.857389000 -1.804357000 0.007175000

8 -2.878345000 1.114780000 -0.000772000

8 -0.635049000 2.502641000 -0.003758000

1 4.136465000 -1.370633000 0.006318000

1 2.019859000 -2.631667000 0.009495000

1 -2.878892000 -2.449687000 -0.877939000

1 -2.876940000 -2.447690000 0.893776000

1 -3.757848000 -1.193643000 0.007418000

1 -2.599413000 2.051299000 -0.003621000

1 1.944255000 2.333933000 -0.003847000

1 4.126452000 1.115715000 -0.000340000

1 -0.386707000 -2.548855000 0.009208000

Cartesian coordinates (in Å) for **QND-OH anion**:

6 3.208310000 -0.843254000 0.005024000

6 2.026627000 -1.554596000 0.006966000

6 0.784562000 -0.877700000 0.004849000

7 -0.395985000 -1.543504000 0.006566000

6 -1.641419000 -0.911842000 0.004702000

6 -1.798930000 0.495731000 0.000522000

6 -0.522411000 1.298972000 -0.001520000

6 0.746894000 0.545243000 0.000688000

6 1.977168000 1.232527000 -0.001191000

6 3.189025000 0.570130000 0.000871000

6 -2.837292000 -1.807244000 0.006800000

8 -2.922362000 1.065405000 -0.001885000

8 -0.528616000 2.536139000 -0.005141000

1 4.155378000 -1.375297000 0.006674000

1 2.037684000 -2.642661000 0.010098000

1 -2.886861000 -2.461980000 -0.879096000

1 -2.884707000 -2.460528000 0.893860000

1 -3.713175000 -1.156885000 0.007099000

1 1.917620000 2.315763000 -0.004316000

1 4.121841000 1.125460000 -0.000658000

1 -0.377798000 -2.551541000 0.009911000

Cartesian coordinates (in Å) for **QND-NH anion**:

6 3.174229000 -0.839327000 -0.094301000

6 1.989076000 -1.539428000 -0.108606000

6 0.731194000 -0.873726000 -0.047416000

7 -0.402157000 -1.617747000 -0.064847000

6 -1.560577000 -0.936491000 -0.003959000

6 -1.673438000 0.449218000 0.074215000

6 -0.522079000 1.310511000 0.096340000

6 0.737068000 0.554465000 0.029946000

6 1.965792000 1.243943000 0.042651000

6 3.170130000 0.572853000 -0.017578000

6 -2.813587000 -1.792162000 -0.024525000

8 -2.914463000 1.082237000 0.134972000

8 -0.572419000 2.557051000 0.164228000

1 4.119447000 -1.374783000 -0.142224000

1 1.969379000 -2.623447000 -0.166760000

1 -2.510956000 -2.837239000 -0.088623000

1 -3.420745000 -1.665444000 0.883097000

1 -3.458655000 -1.567318000 -0.885652000

1 -3.586138000 0.396164000 0.124353000

1 1.913692000 2.326458000 0.101900000

1 4.108710000 1.119942000 -0.006734000

Cartesian coordinates (in Å) for **QND-NH dianion**:

6 3.193860000 -0.850734000 -0.000061000

6 2.000775000 -1.544742000 0.000141000

6 0.733205000 -0.891648000 0.000283000

7 -0.391624000 -1.620245000 0.000265000

6 -1.564994000 -0.932977000 0.000427000

6 -1.761787000 0.493357000 0.000909000

6 -0.521017000 1.316571000 0.000379000

6 0.723966000 0.562683000 0.000197000

6 1.968616000 1.238663000 -0.000034000

6 3.180652000 0.575506000 -0.000170000

6 -2.812906000 -1.788361000 -0.000081000

8 -2.922359000 1.033105000 -0.000497000

8 -0.543012000 2.581807000 -0.000203000

1 4.142371000 -1.389834000 -0.000176000

1 1.979199000 -2.634304000 0.000170000

1 -2.850234000 -2.450006000 -0.881778000

1 -2.851549000 -2.449270000 0.882131000

1 -3.680881000 -1.124442000 -0.000849000

1 1.912681000 2.325308000 -0.000091000

1 4.119037000 1.131249000 -0.000363000

Cartesian coordinates (in Å) for **His^-^ system**:

6 3.282948000 -0.177295000 0.622625000

1 2.574943000 -0.195039000 1.436795000

6 4.563403000 -0.595785000 0.495629000

7 2.901771000 0.336484000 -0.593870000

1 1.938388000 0.778535000 -0.661171000

7 4.923549000 -0.333260000 -0.815526000

6 3.914152000 0.254952000 -1.434063000

1 3.896004000 0.581936000 -2.462861000

7 -2.799066000 1.500323000 0.289825000

1 -3.533091000 2.163621000 0.484563000

6 -1.513094000 1.951532000 0.168601000

6 -1.290319000 3.419547000 0.334718000

1 -0.276927000 3.642005000 0.000275000

1 -2.008773000 4.011880000 -0.250617000

1 -1.381043000 3.741588000 1.382786000

6 -0.445032000 1.096742000 -0.092569000

8 0.773205000 1.580419000 -0.181154000

6 -0.718081000 -0.318665000 -0.278578000

8 0.198084000 -1.145955000 -0.553753000

6 -2.102196000 -0.748358000 -0.125387000

6 -2.456289000 -2.109432000 -0.263369000

1 -1.648835000 -2.800135000 -0.481890000

6 -3.763358000 -2.524993000 -0.125630000

1 -4.021486000 -3.574436000 -0.233534000

6 -4.775721000 -1.582038000 0.157885000

1 -5.806109000 -1.909521000 0.266785000

6 -4.466736000 -0.246416000 0.299126000

1 -5.242951000 0.483993000 0.517620000

6 -3.128998000 0.184301000 0.158193000

6 5.474321000 -1.332169000 1.495506000

1 5.712303000 -2.339956000 1.138543000

1 6.420732000 -0.799294000 1.639198000

1 4.972752000 -1.417256000 2.463644000

Cartesian coordinates (in Å) for **His-H system**:

6 3.241004000 -0.163900000 0.644875000

1 2.560738000 -0.209316000 1.479187000

6 4.523150000 -0.579056000 0.524618000

7 2.863600000 0.345575000 -0.574531000

1 1.449833000 0.940678000 -0.502116000

7 4.888346000 -0.318651000 -0.785545000

1 5.798833000 -0.513932000 -1.174252000

6 3.880096000 0.265345000 -1.410028000

1 3.888611000 0.586337000 -2.439533000

7 -2.844176000 1.495897000 0.281115000

1 -3.574729000 2.165247000 0.469375000

6 -1.558943000 1.950415000 0.164503000

6 -1.340997000 3.419435000 0.328126000

1 -1.377227000 3.721529000 1.382793000

1 -0.360754000 3.689254000 -0.062039000

1 -2.104940000 3.998077000 -0.205492000

6 -0.487360000 1.098010000 -0.089909000

8 0.729989000 1.584961000 -0.174116000

6 -0.755624000 -0.318610000 -0.273689000

8 0.164055000 -1.143982000 -0.542841000

6 -2.139205000 -0.751805000 -0.125632000

6 -2.488889000 -2.114187000 -0.261944000

1 -1.683629000 -2.807880000 -0.476059000

6 -3.795393000 -2.533075000 -0.129012000

1 -4.048502000 -3.582602000 -0.236329000

6 -4.811629000 -1.592285000 0.147760000

1 -5.840592000 -1.921888000 0.252376000

6 -4.506997000 -0.255473000 0.287188000

1 -5.285819000 0.472247000 0.500143000

6 -3.169852000 0.178649000 0.151162000

6 5.431626000 -1.310594000 1.530239000

1 5.665807000 -2.324681000 1.191497000

1 6.372800000 -0.776376000 1.695486000

1 4.906910000 -1.381487000 2.483800000

Cartesian coordinates (in Å) for **His-Asp system**:

6 6.124282000 1.344859000 -0.327037000

1 5.055657000 1.514861000 -0.140683000

1 6.685634000 1.779097000 0.506202000

6 6.419442000 -0.148562000 -0.380648000

8 5.581244000 -0.915472000 -0.951684000

8 7.507161000 -0.521002000 0.075842000

6 1.210560000 -0.821056000 1.262865000

1 0.392589000 -0.952036000 1.952459000

6 2.453345000 -1.355680000 1.244943000

7 1.093952000 -0.028053000 0.146358000

1 -0.164681000 0.696070000 0.162746000

7 3.059508000 -0.876390000 0.095768000

1 4.064709000 -1.001902000 -0.233532000

6 2.228115000 -0.057173000 -0.524946000

1 2.457252000 0.469199000 -1.436905000

7 -4.554262000 1.608185000 0.378338000

1 -5.246543000 2.306547000 0.600632000

6 -3.233597000 1.916660000 0.559157000

6 -2.920411000 3.282165000 1.078389000

1 -1.855709000 3.475839000 0.956416000

1 -3.486627000 4.055209000 0.542257000

1 -3.161649000 3.380590000 2.145504000

6 -2.212282000 1.011087000 0.282657000

8 -0.960967000 1.352491000 0.492084000

6 -2.566845000 -0.293324000 -0.251533000

8 -1.689714000 -1.151068000 -0.558792000

6 -3.987567000 -0.575560000 -0.412049000

6 -4.425472000 -1.827149000 -0.900629000

1 -3.655890000 -2.552361000 -1.141412000

6 -5.766721000 -2.101793000 -1.060981000

1 -6.087928000 -3.067782000 -1.437314000

6 -6.729897000 -1.121785000 -0.734948000

1 -7.786648000 -1.337957000 -0.861198000

6 -6.338955000 0.109835000 -0.255800000

1 -7.076680000 0.867859000 -0.003544000

6 -4.965785000 0.396168000 -0.090450000

6 6.520506000 2.053550000 -1.635610000

1 5.972509000 1.627856000 -2.481989000

1 6.306960000 3.128157000 -1.594621000

1 7.590423000 1.926013000 -1.829161000

6 3.108519000 -2.384778000 2.184869000

1 3.260483000 -3.341284000 1.675535000

1 4.082384000 -2.037772000 2.542152000

1 2.454143000 -2.546771000 3.044119000

Cartesian coordinates (in Å) for **His-Asp-Trp system**:

6 6.308835000 -2.319958000 -0.050800000

8 5.546045000 -1.526992000 0.494123000

7 7.500513000 -2.643702000 0.432213000

1 8.065368000 -3.283925000 -0.103355000

6 -7.149894000 -2.274477000 -0.360823000

1 -6.063892000 -2.187681000 -0.225172000

1 -7.538770000 -2.922559000 0.430944000

6 -7.808190000 -0.906916000 -0.231522000

8 -7.219197000 0.102883000 -0.731739000

8 -8.927071000 -0.871695000 0.294882000

6 -2.844814000 0.879856000 1.336436000

1 -2.047157000 1.140199000 2.013149000

6 -4.180278000 1.082353000 1.414990000

7 -2.598241000 0.264939000 0.132804000

1 -1.179624000 -0.112243000 0.031265000

7 -4.711954000 0.590409000 0.234762000

1 -5.731968000 0.494812000 -0.048105000

6 -3.739483000 0.078258000 -0.500076000

1 -3.881180000 -0.387651000 -1.461389000

7 3.283444000 0.092110000 0.017105000

1 4.137664000 -0.444497000 0.136545000

6 2.092509000 -0.557002000 0.196911000

6 2.156659000 -2.004590000 0.561142000

1 1.182687000 -2.326605000 0.927581000

1 2.409496000 -2.628956000 -0.307373000

1 2.916584000 -2.193194000 1.327009000

6 0.864956000 0.085712000 0.056280000

8 -0.248887000 -0.581050000 0.257329000

6 0.855134000 1.486269000 -0.332015000

8 -0.222799000 2.122593000 -0.512668000

6 2.150491000 2.133364000 -0.496955000

6 2.236888000 3.499536000 -0.847748000

1 1.299846000 4.029241000 -0.981210000

6 3.457261000 4.119192000 -1.011479000

1 3.507279000 5.169750000 -1.280764000

6 4.649747000 3.385060000 -0.828584000

1 5.611374000 3.874222000 -0.956571000

6 4.603304000 2.050945000 -0.486115000

1 5.514160000 1.477243000 -0.338684000

6 3.355229000 1.411464000 -0.317904000

6 8.156351000 -1.929832000 1.537315000

1 7.419959000 -1.259434000 1.978239000

1 8.503962000 -2.636733000 2.294673000

1 9.005846000 -1.346705000 1.169978000

6 5.926182000 -3.051287000 -1.351337000

1 6.640043000 -3.828678000 -1.633905000

1 4.936333000 -3.494325000 -1.227096000

1 5.861252000 -2.315967000 -2.157556000

6 -7.430586000 -2.917054000 -1.731919000

1 -7.053102000 -2.278923000 -2.537006000

1 -6.955397000 -3.900976000 -1.822973000

1 -8.507883000 -3.043946000 -1.880146000

6 -5.016465000 1.806792000 2.486326000

1 -5.432959000 2.740416000 2.095773000

1 -5.849862000 1.187938000 2.831682000

1 -4.374129000 2.040857000 3.338115000

Cartesian coordinates (in Å) for **wt system**:

7 1.025773000 -1.427297000 -5.572996000

1 0.340653000 -1.041306000 -4.914688000

6 1.431632000 -0.545122000 -6.689532000

6 0.337300000 0.468096000 -6.995136000

6 2.700700000 0.196120000 -6.343266000

8 2.752800000 1.257036000 -5.773815000

7 5.428124000 -1.581227000 -1.596364000

1 6.062954000 -1.113209000 -0.970003000

6 5.531673000 -1.603491000 -3.018448000

6 4.330803000 -2.350400000 -1.260265000

7 3.727891000 -2.810462000 -2.419227000

6 4.502165000 -2.370566000 -3.490605000

7 0.180549000 -7.086116000 0.133975000

1 0.486596000 -6.994385000 -0.830003000

6 0.132014000 -5.859568000 0.841287000

6 1.602292000 -5.277420000 1.222734000

7 1.119762000 -4.180186000 3.398349000

6 1.547836000 -4.042157000 2.096224000

6 1.186306000 -2.874847000 3.958879000

7 1.688106000 -1.990357000 2.894520000

1 1.774107000 -0.988696000 2.963439000

6 1.905129000 -2.767662000 1.797831000

6 -0.600708000 -4.769248000 0.120112000

8 -0.373351000 -4.554041000 -1.034531000

7 -1.541962000 -4.098931000 0.845254000

1 -1.651571000 -4.339555000 1.818976000

6 -2.496420000 -3.110329000 0.297695000

6 -1.876175000 -1.708331000 0.088419000

8 -2.766993000 -0.796445000 0.336863000

1 -2.487066000 0.137909000 0.077436000

6 -3.273093000 -3.606403000 -0.914952000

8 -3.814773000 -4.692452000 -0.881963000

7 -3.289287000 -2.868337000 -2.031845000

1 -2.861804000 -1.953905000 -2.033417000

6 -4.035338000 -3.184739000 -3.259024000

6 -4.045131000 -1.969909000 -4.224062000

7 -3.631397000 0.173408000 -2.923431000

6 -4.502021000 -0.746893000 -3.420659000

6 -4.372805000 1.024587000 -2.163794000

7 -5.680306000 0.631995000 -2.175256000

1 -6.355005000 1.151570000 -1.614569000

6 -5.809721000 -0.505975000 -2.965591000

6 -3.560208000 -4.356160000 -4.050657000

8 -4.163749000 -4.762308000 -5.015922000

6 -2.149055000 -1.487138000 6.450956000

8 -3.037991000 -1.493428000 7.341537000

8 -1.288223000 -0.486297000 6.299526000

6 0.122713000 2.436886000 2.848790000

6 0.049595000 2.031549000 4.215985000

7 -0.761403000 1.835203000 2.321854000

7 -1.008954000 1.132482000 4.264536000

1 -1.219060000 0.485287000 5.147810000

6 -1.532117000 0.975347000 3.036413000

7 -0.259536000 3.594165000 -2.786569000

1 0.518280000 3.531082000 -3.429457000

6 -0.396626000 2.592870000 -1.842155000

6 0.660279000 1.531459000 -1.836192000

1 1.655160000 1.949960000 -2.036995000

1 0.663374000 1.024670000 -0.870432000

1 0.445995000 0.768711000 -2.597095000

6 -1.494166000 2.561016000 -0.982029000

8 -1.611629000 1.575240000 -0.066018000

1 -1.044360000 1.855451000 1.258565000

6 -2.499609000 3.612682000 -1.069645000

8 -3.556312000 3.607972000 -0.335740000

6 -2.284285000 4.662279000 -2.066061000

6 -3.203800000 5.727646000 -2.204427000

1 -4.057764000 5.734331000 -1.536569000

6 -3.016061000 6.716755000 -3.156578000

1 -3.730740000 7.527934000 -3.252159000

6 -1.891016000 6.666256000 -4.012262000

1 -1.746314000 7.437986000 -4.762324000

6 -0.969979000 5.636667000 -3.902040000

1 -0.104880000 5.591377000 -4.558536000

6 -1.157357000 4.629809000 -2.927084000

8 0.741200000 -2.307378000 -2.111134000

1 0.318803000 -3.150277000 -1.820554000

1 1.668416000 -2.469109000 -2.398533000

8 -6.110329000 2.600555000 -0.201068000

1 -5.265033000 3.109342000 -0.328740000

1 -6.039005000 2.150996000 0.661427000

8 -0.889964000 -0.788148000 -3.605161000

1 -0.360541000 -1.305270000 -2.930593000

1 -1.681956000 -0.303104000 -3.266250000

6 6.696227000 -0.857106000 -3.694757000

1 6.665609000 0.215455000 -3.469539000

1 7.667240000 -1.253739000 -3.373490000

1 6.624774000 -0.972650000 -4.779492000

6 0.892746000 2.543354000 5.389930000

1 0.377505000 3.346596000 5.930634000

1 1.096877000 1.742752000 6.108401000

1 1.840403000 2.935348000 5.009308000

6 -1.930347000 -2.831554000 5.672965000

1 -1.647877000 -2.665318000 4.627278000

1 -1.118468000 -3.389859000 6.156033000

1 -2.845235000 -3.424768000 5.728098000

1 -0.655487000 -7.653747000 0.207993000

1 0.789519000 3.141090000 2.381234000

1 -2.309662000 0.337837000 2.658194000

1 2.169795000 -2.342263000 0.842734000

1 0.877449000 -2.530507000 4.930182000

1 4.214871000 -2.576381000 -4.507117000

1 3.985179000 -2.531023000 -0.258741000

1 0.619696000 1.155495000 -7.803712000

1 -0.586270000 -0.045014000 -7.281012000

1 0.129493000 1.066575000 -6.103391000

1 -4.007435000 1.833211000 -1.564916000

1 -6.745650000 -1.009220000 -3.133510000

1 -3.035591000 -1.786311000 -4.606094000

1 -4.714268000 -2.199997000 -5.055135000

1 -5.077719000 -3.399909000 -2.979735000

1 -2.599129000 -4.803745000 -3.731396000

1 -3.243293000 -2.966488000 1.087322000

1 -0.343125000 -6.048689000 1.810946000

1 2.128276000 -5.066128000 0.283368000

1 2.120373000 -6.090876000 1.738694000

1 1.644837000 -1.166491000 -7.570738000

1 3.639619000 -0.359577000 -6.573801000

1 0.864372000 -2.393633000 -5.815820000

1 -1.408402000 -1.646641000 -0.907879000

1 -1.000150000 -1.671781000 0.774041000

Cartesian coordinates (in Å) for **D126A system**:

7 1.027731000 -1.440561000 -5.558266000

1 0.304112000 -1.091470000 -4.920612000

6 1.432614000 -0.557994000 -6.684431000

6 0.337458000 0.453446000 -6.987630000

6 2.704325000 0.183240000 -6.338757000

8 2.743346000 1.250571000 -5.752513000

7 5.422758000 -1.584448000 -1.573858000

1 6.069115000 -1.141228000 -0.941176000

6 5.546307000 -1.612424000 -3.027221000

6 4.349350000 -2.366617000 -1.265064000

7 3.727113000 -2.816355000 -2.399969000

6 4.515415000 -2.384934000 -3.495994000

7 0.187578000 -7.091087000 0.156029000

1 0.507030000 -7.054376000 -0.804234000

6 0.160728000 -5.886599000 0.844626000

6 1.620901000 -5.299575000 1.220463000

7 1.115618000 -4.183749000 3.415246000

6 1.582554000 -4.066411000 2.098804000

6 1.219439000 -2.896251000 3.959830000

7 1.674453000 -1.987816000 2.907489000

1 1.795091000 -0.989315000 2.973734000

6 1.933200000 -2.791772000 1.796442000

6 -0.576614000 -4.791484000 0.126465000

8 -0.389122000 -4.552045000 -1.006645000

7 -1.542124000 -4.104258000 0.861930000

1 -1.670346000 -4.368495000 1.826595000

6 -2.469770000 -3.128161000 0.308561000

6 -1.857977000 -1.730139000 0.101546000

8 -2.803826000 -0.800735000 0.338546000

1 -2.589368000 0.085285000 -0.017459000

6 -3.251775000 -3.631959000 -0.900586000

8 -3.830906000 -4.704289000 -0.873273000

7 -3.289474000 -2.885730000 -2.023807000

1 -2.754337000 -2.032580000 -2.081692000

6 -4.019646000 -3.207722000 -3.241498000

6 -4.034228000 -1.996280000 -4.202615000

7 -3.637785000 0.155952000 -2.918131000

6 -4.489679000 -0.767743000 -3.401129000

6 -4.364140000 0.989710000 -2.148262000

7 -5.679588000 0.614837000 -2.171924000

1 -6.418006000 1.103163000 -1.690776000

6 -5.794934000 -0.534741000 -2.950743000

6 -3.549659000 -4.383913000 -4.036418000

8 -4.157337000 -4.792463000 -5.013330000

6 0.153372000 2.405573000 2.864066000

6 0.077645000 1.997912000 4.225526000

7 -0.782117000 1.826091000 2.327263000

7 -1.032682000 1.127079000 4.274412000

1 -1.344968000 0.665724000 5.115713000

6 -1.504956000 0.969019000 3.040446000

7 0.849425000 2.280363000 -3.466020000

1 1.536476000 2.051368000 -4.183570000

6 0.915470000 1.627863000 -2.259627000

6 2.067320000 0.705075000 -2.007625000

1 1.697801000 -0.302073000 -1.789436000

1 2.738681000 0.653505000 -2.869086000

1 2.636355000 1.042137000 -1.132949000

6 -0.079179000 1.829763000 -1.319679000

8 -0.005732000 1.141113000 -0.128496000

1 -0.541059000 1.546381000 0.641437000

6 -1.208838000 2.670293000 -1.580840000

8 -2.147525000 2.753592000 -0.694596000

6 -1.221299000 3.374435000 -2.854544000

6 -2.269396000 4.259636000 -3.206723000

1 -3.085774000 4.402671000 -2.506057000

6 -2.262713000 4.908556000 -4.430766000

1 -3.071652000 5.582396000 -4.692257000

6 -1.207921000 4.689757000 -5.346269000

1 -1.209130000 5.201149000 -6.303638000

6 -0.173197000 3.822992000 -5.035256000

1 0.638264000 3.642939000 -5.733414000

6 -0.171869000 3.156282000 -3.788634000

8 0.848666000 -2.403832000 -2.119925000

1 0.389647000 -3.225866000 -1.830976000

1 1.778501000 -2.599497000 -2.394725000

8 -4.566196000 3.888812000 -0.665996000

1 -3.624449000 3.564916000 -0.595415000

1 -4.706639000 4.633428000 -0.054379000

8 -0.910401000 -0.965353000 -3.568951000

1 -0.292698000 -1.383362000 -2.905503000

1 -1.606735000 -0.364086000 -3.221384000

6 6.717860000 -0.874524000 -3.712262000

1 6.689065000 0.200304000 -3.500468000

1 7.685635000 -1.270665000 -3.383203000

1 6.646670000 -1.007839000 -4.794762000

6 0.924649000 2.516567000 5.400100000

1 0.338541000 3.134152000 6.092447000

1 1.391383000 1.712007000 5.982750000

1 1.721309000 3.140008000 4.986159000

1 -0.595753000 -7.714736000 0.288198000

1 0.848948000 3.087914000 2.400757000

1 -2.314493000 0.351481000 2.693302000

1 2.200143000 -2.358964000 0.845950000

1 0.955891000 -2.584127000 4.954598000

1 4.228158000 -2.607029000 -4.508620000

1 4.004356000 -2.551829000 -0.263213000

1 0.636546000 1.172008000 -7.761064000

1 -0.565231000 -0.063227000 -7.328098000

1 0.076009000 1.014176000 -6.086587000

1 -4.004107000 1.851335000 -1.619653000

1 -6.727575000 -1.042765000 -3.122391000

1 -3.027016000 -1.812092000 -4.589088000

1 -4.706784000 -2.223206000 -5.031611000

1 -5.061221000 -3.428390000 -2.962487000

1 -2.595521000 -4.843010000 -3.715982000

1 -3.232466000 -2.979939000 1.084230000

1 -0.314658000 -6.058697000 1.818082000

1 2.146953000 -5.081848000 0.282358000

1 2.146739000 -6.114323000 1.727440000

1 1.642635000 -1.178468000 -7.566063000

1 3.652293000 -0.337105000 -6.586603000

1 0.966542000 -2.425553000 -5.766781000

1 -1.011873000 -1.650192000 0.806397000

1 -1.384668000 -1.643606000 -0.884388000

Cartesian coordinates (in Å) for **H251A system**:

7 0.082417000 0.090653000 -0.139355000

6 1.551736000 0.088282000 -0.151006000

6 2.086952000 1.533796000 -0.155363000

6 2.155370000 -0.665005000 1.042775000

8 1.634663000 -0.564030000 2.175892000

6 0.660294000 -4.960359000 3.282426000

6 -0.585223000 -4.265512000 2.786039000

7 -1.753005000 -4.271819000 3.510818000

6 -2.700797000 -3.602060000 2.859899000

7 -2.172072000 -3.178597000 1.702005000

6 -0.856841000 -3.594482000 1.640648000

6 -7.536281000 -1.102385000 0.132177000

6 -6.862266000 -2.217859000 0.919270000

6 -6.532104000 -1.778801000 2.313286000

7 -7.478541000 -1.685367000 3.300371000

6 -6.929829000 -1.235898000 4.417376000

7 -5.649242000 -0.957726000 4.167176000

6 -5.378716000 -1.319201000 2.861188000

6 -6.728638000 0.183001000 0.254412000

7 -7.258121000 1.121461000 1.039627000

6 -6.634679000 2.411356000 1.324935000

6 -5.633330000 2.276307000 2.489388000

8 -5.117015000 3.530461000 2.905699000

6 -6.039676000 3.079156000 0.068696000

7 -4.664065000 3.285229000 0.052080000

6 -4.021333000 3.990179000 -1.059005000

6 -2.783916000 4.733839000 -0.548360000

6 -3.119646000 5.784490000 0.479448000

7 -3.750057000 6.978806000 0.155962000

6 -3.962856000 7.659301000 1.276854000

7 -3.478286000 6.976927000 2.289927000

6 -2.968699000 5.821371000 1.822159000

8 -3.825395000 2.189135000 4.905744000

6 -2.589309000 1.849703000 4.935071000

6 -1.507372000 2.833550000 4.922420000

6 -1.781240000 4.220134000 4.959992000

6 -0.756495000 5.153290000 4.912678000

6 0.585658000 4.719023000 4.830347000

6 0.888733000 3.366188000 4.805960000

6 -0.154125000 2.413811000 4.858741000

7 0.114211000 1.059724000 4.851689000

6 -0.865775000 0.088953000 4.931443000

6 -2.196085000 0.458884000 4.976353000

8 -3.125730000 -0.546405000 5.056703000

1 -5.685213000 -0.119780000 0.517759000

1 3.183289000 1.591860000 -0.193610000

1 1.745153000 2.054159000 0.745900000

1 1.892137000 -0.418983000 -1.063942000

1 -0.317253000 0.268390000 0.775949000

1 1.483624000 -4.853397000 2.568877000

1 -0.242160000 -3.329265000 0.795354000

1 -2.618722000 -2.622559000 0.989662000

1 -3.702806000 -3.418266000 3.200154000

1 -5.927048000 -2.497676000 0.420829000

1 -7.482263000 -3.126590000 0.931175000

1 -4.402647000 -1.183516000 2.432773000

1 -4.090895000 -0.314025000 4.916764000

1 -7.441073000 -1.075324000 5.349719000

1 -8.560349000 -0.929027000 0.494618000

1 -4.822703000 1.597768000 2.183332000

1 -6.155524000 1.780035000 3.319848000

1 -4.598695000 3.364558000 3.734170000

1 -7.436003000 3.066841000 1.688197000

1 -7.831458000 0.803037000 1.810429000

1 -2.243491000 5.172728000 -1.399307000

1 -2.104159000 4.008826000 -0.084295000

1 -2.528424000 5.082201000 2.468016000

1 -4.437047000 8.624633000 1.328645000

1 -4.714440000 4.726136000 -1.520337000

1 -2.815924000 4.535687000 5.027987000

1 -0.987333000 6.212488000 4.928520000

1 1.387723000 5.449223000 4.791346000

1 1.920398000 3.028364000 4.755573000

1 1.065734000 0.748323000 4.715521000

1 3.089569000 -1.225438000 0.883953000

1 -0.336272000 0.645755000 -0.874545000

1 -8.432757000 -1.996337000 3.195602000

1 -4.028571000 7.244575000 -0.775592000

1 0.469217000 -6.028193000 3.436252000

1 0.977256000 -4.542706000 4.244406000

1 1.697243000 2.061252000 -1.032564000

6 -0.398962000 -1.333622000 4.972309000

1 0.295925000 -1.540349000 4.149472000

1 0.122293000 -1.543359000 5.917233000

1 -1.243585000 -2.016864000 4.897271000

1 -7.637126000 -1.406311000 -0.916398000

1 -6.601299000 0.574911000 -0.773510000

1 -6.599657000 4.036305000 -0.069560000

1 -3.739633000 3.291067000 -1.862293000

1 -6.338622000 2.488428000 -0.810758000

1 -4.344421000 3.641468000 0.953436000

Cartesian coordinates (in Å) for **wt QM region at 300 ps**:

| N | 0.4692137 | 4.09347093 | -5.229058854 |
| --- | --- | --- | --- |
| H | -0.4749656 | 4.1117584 | -4.867176142 |
| C | 0.81167817 | 4.87796647 | -6.404674518 |
| H | 1.06020478 | 4.2497215 | -7.282925511 |
| C | 1.95845066 | 5.89572694 | -6.174952642 |
| O | 1.97543971 | 6.57692106 | -5.156573324 |
| C | 6.33870727 | 5.24999301 | -4.850777063 |
| H | 7.22242372 | 5.81165804 | -5.201910442 |
| C | 6.21149628 | 5.46410586 | -3.331589746 |
| H | 5.91130802 | 6.51664042 | -3.169797443 |
| H | 7.20626317 | 5.39249707 | -2.851313309 |
| N | 5.2320329 | 4.53110685 | -1.208556757 |
| H | 5.90914111 | 5.02306539 | -0.601730478 |
| C | 5.26596612 | 4.57151392 | -2.585191433 |
| C | 4.29809713 | 3.60887199 | -0.840801844 |
| H | 4.07228873 | 3.36858347 | 0.199183461 |
| N | 3.7213961 | 3.06234532 | -1.892382584 |
| C | 4.3196188 | 3.65194477 | -2.985402335 |
| H | 4.04432914 | 3.3850798 | -4.005143507 |
| C | 0.16395562 | -0.1275653 | 1.593533632 |
| H | -0.0616867 | -0.1795073 | 2.671715222 |
| C | 1.56229342 | 0.54428777 | 1.435161203 |
| H | 1.81087095 | 0.64946676 | 0.363589781 |
| H | 2.33106634 | -0.0914706 | 1.903557553 |
| N | 1.50781912 | 2.07809245 | 3.447210543 |
| C | 1.53251546 | 1.90246598 | 2.076867092 |
| C | 1.14988371 | 3.33263706 | 3.647180992 |
| H | 0.9976302 | 3.81416493 | 4.613770554 |
| N | 0.96002383 | 3.99462323 | 2.473108457 |
| H | 0.53486022 | 4.91926966 | 2.372259646 |
| C | 1.20551609 | 3.09271174 | 1.460487466 |
| H | 1.05759021 | 3.32457053 | 0.409366228 |
| C | -0.8651169 | 0.76058724 | 0.892809795 |
| O | -0.8458036 | 0.96715566 | -0.325761536 |
| N | -1.7919585 | 1.35124489 | 1.695746716 |
| H | -1.7829248 | 1.16177429 | 2.702581597 |
| C | -2.7588872 | 2.32016802 | 1.213311958 |
| H | -3.5267919 | 2.41540561 | 1.995765203 |
| C | -2.1073644 | 3.69435901 | 1.056299112 |
| H | -1.3252062 | 3.64149937 | 0.281529665 |
| H | -1.623649 | 3.93636757 | 2.014661208 |
| O | -3.0959764 | 4.6430954 | 0.71996375 |
| H | -2.7001932 | 5.54523522 | 0.579998587 |
| C | -3.4884843 | 1.7643834 | -0.018851755 |
| O | -3.8918046 | 0.60237887 | -0.042564809 |
| N | -3.6269591 | 2.61469957 | -1.076017188 |
| H | -3.4813348 | 3.60978064 | -0.862124798 |
| C | -4.3769465 | 2.29920567 | -2.281160811 |
| H | -5.3244568 | 1.8012446 | -2.003030874 |
| C | -4.695593 | 3.58103685 | -3.080421841 |
| H | -5.1553349 | 3.31158891 | -4.047264735 |
| H | -3.7661537 | 4.13701071 | -3.303913592 |
| N | -5.1328841 | 5.63801783 | -1.707723341 |
| C | -5.6079553 | 4.50419578 | -2.320140414 |
| C | -6.1515074 | 6.15283157 | -1.048328167 |
| H | -6.1211802 | 7.04174472 | -0.415575827 |
| N | -7.2762967 | 5.40785854 | -1.213742136 |
| H | -8.1781543 | 5.5311763 | -0.74437805 |
| C | -6.9506452 | 4.34901699 | -2.027614797 |
| H | -7.6917838 | 3.60919891 | -2.317559533 |
| C | -3.2078838 | 2.29331267 | 6.343782311 |
| H | -4.0386144 | 2.22426557 | 7.079227108 |
| C | -2.2514647 | 3.39496955 | 6.897483402 |
| H | -1.5425918 | 3.76413137 | 6.136429932 |
| H | -1.6274039 | 2.95037538 | 7.700677631 |
| C | -2.9688796 | 4.57163447 | 7.594500493 |
| O | -2.7684402 | 5.7695333 | 7.19250273 |
| O | -3.7367627 | 4.31571276 | 8.539713256 |
| C | 0.67097388 | 11.5654637 | 6.03865476 |
| H | -0.0510548 | 10.7914471 | 6.352792745 |
| C | 1.35978719 | 11.1028079 | 4.730297496 |
| H | 2.04580223 | 10.2473403 | 4.915979931 |
| H | 2.02370756 | 11.898369 | 4.33691392 |
| C | 0.40118108 | 10.6977177 | 3.636080486 |
| C | -0.9684454 | 10.4355261 | 3.754437853 |
| H | -1.6042051 | 10.4697584 | 4.638377929 |
| N | -1.5114524 | 10.1896038 | 2.52833388 |
| H | -2.5071667 | 9.96991114 | 2.385799829 |
| C | -0.5219822 | 10.2482578 | 1.570154587 |
| C | 0.69607203 | 10.5759663 | 2.235805177 |
| C | 1.87576302 | 10.7063032 | 1.475749878 |
| H | 2.8145387 | 10.9788809 | 1.963446738 |
| C | 1.82628015 | 10.4890838 | 0.101287304 |
| H | 2.73798502 | 10.5690615 | -0.496440546 |
| C | -0.5793448 | 10.042174 | 0.190068946 |
| H | -1.5213158 | 9.79408704 | -0.299963197 |
| C | 0.60816492 | 10.1637536 | -0.534655563 |
| H | 0.59587443 | 9.99655237 | -1.615850387 |
| C | 0.53805346 | 7.09084216 | 7.400663855 |
| H | -0.3526706 | 6.60901116 | 7.841072197 |
| C | 0.08224789 | 8.00651673 | 6.225746568 |
| H | 0.92899277 | 8.59291894 | 5.829374722 |
| H | -0.6373873 | 8.74075991 | 6.636602746 |
| N | -1.6576812 | 6.5683598 | 5.089328101 |
| H | -2.1686153 | 6.17933625 | 6.015616914 |
| C | -0.5637625 | 7.35836708 | 5.049896198 |
| C | -1.8303536 | 6.06255121 | 3.837869919 |
| H | -2.630456 | 5.35216839 | 3.60584402 |
| N | -0.8954752 | 6.50100566 | 2.971271024 |
| C | -0.1109423 | 7.30581664 | 3.684766984 |
| H | 0.75501123 | 7.81505856 | 3.264252831 |
| N | 0.03770287 | 7.10345074 | -3.270706729 |
| H | 0.66460644 | 7.01592423 | -4.082411201 |
| C | 0.42500142 | 6.44305255 | -2.145915635 |
| C | 1.73954644 | 5.71889583 | -2.143893699 |
| H | 2.41167555 | 6.12419348 | -1.363479626 |
| H | 1.6178357 | 4.63750896 | -1.957875162 |
| H | 2.27387584 | 5.83139206 | -3.09216832 |
| C | -0.3752461 | 6.45574537 | -1.024248934 |
| O | 0.02216965 | 5.82836492 | 0.120806043 |
| H | 0.94897184 | 5.55248217 | 0.03130988 |
| C | -1.6462092 | 7.13558962 | -0.996833704 |
| O | -2.3772389 | 7.13359514 | 0.018735116 |
| C | -1.9955063 | 7.85107842 | -2.226867517 |
| C | -3.1940214 | 8.5967744 | -2.310502915 |
| H | -3.8605335 | 8.5754972 | -1.44542548 |
| C | -3.5044212 | 9.31661294 | -3.451785914 |
| H | -4.4346995 | 9.89101882 | -3.505184303 |
| C | -2.6260854 | 9.29130301 | -4.560121718 |
| H | -2.8685734 | 9.83962512 | -5.474665067 |
| C | -1.4527544 | 8.55659641 | -4.515415403 |
| H | -0.7709042 | 8.55514343 | -5.372699725 |
| C | -1.1226831 | 7.82932094 | -3.345552339 |
| H | 1.0067047 | 3.28312682 | -4.995781444 |
| H | -0.0856588 | 5.43052521 | -6.683121009 |
| H | 2.73308223 | 6.02333188 | -6.93108109 |
| H | 5.49300164 | 5.54412756 | -5.47233491 |
| H | 6.54763497 | 4.21436223 | -5.118891674 |
| H | 0.12035168 | -1.1118961 | 1.127423119 |
| H | -3.8957786 | 1.56125142 | -2.923008346 |
| H | -2.763957 | 1.29855278 | 6.381849489 |
| H | -3.7028389 | 2.62520708 | 5.431130767 |
| H | 0.10247015 | 12.4853935 | 5.90227285 |
| H | 1.38090928 | 11.5643991 | 6.865732699 |
| H | 1.01372954 | 7.73751496 | 8.13796561 |
| H | 1.12570147 | 6.25990319 | 7.010436287 |

Cartesian coordinates (in Å) for **wt QM region at 400 ps**:

| N | 0.73458343 | 4.35259083 | -5.011485789 |
| --- | --- | --- | --- |
| H | -0.1195139 | 4.57304914 | -4.519272736 |
| C | 1.15098636 | 5.11504051 | -6.177658044 |
| H | 1.43183332 | 4.45284618 | -7.017650599 |
| C | 2.30859463 | 6.11195698 | -5.932506735 |
| O | 2.31710246 | 6.80558892 | -4.92150987 |
| C | 6.56267499 | 5.20925615 | -4.430337428 |
| H | 7.48906004 | 5.72365707 | -4.742826302 |
| C | 6.38982206 | 5.43421863 | -2.914852663 |
| H | 6.11005571 | 6.49484407 | -2.76869617 |
| H | 7.36573228 | 5.34239501 | -2.402145986 |
| N | 5.36938691 | 4.47001991 | -0.819637685 |
| H | 6.06338916 | 4.90148795 | -0.184771176 |
| C | 5.4076122 | 4.55936754 | -2.194955106 |
| C | 4.39648493 | 3.57592991 | -0.486342882 |
| H | 4.15888185 | 3.31303825 | 0.544803039 |
| N | 3.80055035 | 3.09031494 | -1.557260277 |
| C | 4.4268584 | 3.69204145 | -2.627826764 |
| H | 4.14660148 | 3.46971546 | -3.65721739 |
| C | 0.12846658 | -0.0112249 | 1.74062739 |
| H | -0.1145032 | -0.0418591 | 2.816199692 |
| C | 1.53771232 | 0.64617119 | 1.565250306 |
| H | 1.82173922 | 0.63671748 | 0.497273611 |
| H | 2.28965113 | 0.05523768 | 2.115768909 |
| N | 1.73636912 | 2.42356428 | 3.37304402 |
| C | 1.5353582 | 2.06954231 | 2.051952558 |
| C | 1.39463673 | 3.69842072 | 3.475212203 |
| H | 1.40037678 | 4.29309705 | 4.391266078 |
| N | 1.00302959 | 4.20322218 | 2.279975356 |
| H | 0.50266812 | 5.08859136 | 2.149621406 |
| C | 1.10077631 | 3.17863481 | 1.359896932 |
| H | 0.80348687 | 3.27939713 | 0.321304863 |
| C | -0.889594 | 0.85509467 | 0.996343651 |
| O | -0.812202 | 1.07729479 | -0.216921289 |
| N | -1.8645269 | 1.42795474 | 1.754590946 |
| H | -1.9470988 | 1.18511975 | 2.746855975 |
| C | -2.8110036 | 2.39911838 | 1.233284095 |
| H | -3.61834 | 2.47731565 | 1.976107199 |
| C | -2.1552289 | 3.77747885 | 1.130349347 |
| H | -1.3353192 | 3.73481941 | 0.398061613 |
| H | -1.7268172 | 4.0082456 | 2.11834112 |
| O | -3.1150991 | 4.73753719 | 0.744611974 |
| H | -2.6834082 | 5.61329882 | 0.553127697 |
| C | -3.4752885 | 1.86387591 | -0.042477353 |
| O | -3.903306 | 0.71186966 | -0.095521438 |
| N | -3.531409 | 2.72012967 | -1.102528981 |
| H | -3.3830866 | 3.71080477 | -0.872920495 |
| C | -4.2126383 | 2.4329385 | -2.354685 |
| H | -5.2027302 | 1.98602914 | -2.143962559 |
| C | -4.4068061 | 3.72747449 | -3.172463747 |
| H | -4.8207527 | 3.4833329 | -4.165592317 |
| H | -3.4343476 | 4.22625964 | -3.338779263 |
| N | -4.7978708 | 5.80389333 | -1.819278377 |
| C | -5.3073927 | 4.70521128 | -2.467325085 |
| C | -5.817572 | 6.38282771 | -1.22072531 |
| H | -5.7652843 | 7.25950313 | -0.570584482 |
| N | -6.9797177 | 5.71934021 | -1.464813165 |
| H | -7.8986923 | 5.89547111 | -1.050696197 |
| C | -6.6729466 | 4.63875859 | -2.257797276 |
| H | -7.4396068 | 3.9397674 | -2.579927991 |
| C | -3.6315343 | 2.34447684 | 6.188133497 |
| H | -4.5110439 | 2.29645538 | 6.863584339 |
| C | -2.7158842 | 3.44129339 | 6.794348434 |
| H | -1.947281 | 3.80507012 | 6.090066214 |
| H | -2.1493745 | 3.01162588 | 7.648566182 |
| C | -3.4661543 | 4.63590344 | 7.41682077 |
| O | -3.0218591 | 5.81811629 | 7.239877243 |
| O | -4.4792686 | 4.39385781 | 8.10499662 |
| C | 0.6473807 | 11.7785883 | 6.56597238 |
| H | -0.0584341 | 11.0053105 | 6.924015606 |
| C | 1.34266756 | 11.2695624 | 5.273014328 |
| H | 1.99598365 | 10.3959934 | 5.489955399 |
| H | 2.04162272 | 12.0395737 | 4.884341443 |
| C | 0.44136145 | 10.8712311 | 4.131183265 |
| C | -0.9582367 | 10.717023 | 4.12536297 |
| H | -1.6656863 | 10.8340314 | 4.94533277 |
| N | -1.3935687 | 10.4466132 | 2.871119215 |
| H | -2.3902038 | 10.3777564 | 2.588148076 |
| C | -0.3108274 | 10.3746744 | 2.011924716 |
| C | 0.85807367 | 10.6632228 | 2.772598248 |
| C | 2.10810079 | 10.6963906 | 2.125535339 |
| H | 3.01315069 | 10.9492282 | 2.685371863 |
| C | 2.17210839 | 10.4064138 | 0.761590278 |
| H | 3.13301514 | 10.4284812 | 0.241619628 |
| C | -0.251903 | 10.0560693 | 0.6562925 |
| H | -1.1568271 | 9.81092104 | 0.097736535 |
| C | 1.00766181 | 10.0779329 | 0.039419942 |
| H | 1.09077836 | 9.84093075 | -1.025654619 |
| C | 0.3597313 | 6.92137105 | 7.555904922 |
| H | -0.6196458 | 6.54600446 | 7.902525946 |
| C | 0.13980148 | 7.87323535 | 6.344169048 |
| H | 1.09442456 | 8.30615849 | 5.989368363 |
| H | -0.4767411 | 8.72583402 | 6.688712049 |
| N | -1.6654903 | 6.55428435 | 5.182264375 |
| H | -2.2204502 | 6.2293727 | 6.067943239 |
| C | -0.5369712 | 7.30115545 | 5.146662617 |
| C | -1.9048111 | 6.11663417 | 3.918302193 |
| H | -2.7390297 | 5.44467944 | 3.686380367 |
| N | -0.983788 | 6.55995225 | 3.045890796 |
| C | -0.1341708 | 7.29567418 | 3.778188702 |
| H | 0.73513045 | 7.79305067 | 3.348354665 |
| N | 0.33632989 | 7.37531433 | -3.134343675 |
| H | 1.01086388 | 7.32093353 | -3.91061995 |
| C | 0.64762854 | 6.6517519 | -2.026353942 |
| C | 1.97304019 | 5.94743051 | -1.95368335 |
| H | 2.585055 | 6.35958673 | -1.128479053 |
| H | 1.86168911 | 4.86179783 | -1.78516936 |
| H | 2.56174967 | 6.07897502 | -2.865721262 |
| C | -0.2387244 | 6.58608499 | -0.972421812 |
| O | 0.08114552 | 5.87167587 | 0.14584147 |
| H | 1.00118502 | 5.56805642 | 0.071132472 |
| C | -1.5081264 | 7.26622179 | -0.988368211 |
| O | -2.3187199 | 7.18552928 | -0.037975586 |
| C | -1.7567594 | 8.07887008 | -2.181045074 |
| C | -2.9322253 | 8.85463864 | -2.291556602 |
| H | -3.6363422 | 8.81476391 | -1.457846301 |
| C | -3.1721741 | 9.62790407 | -3.413875824 |
| H | -4.0831225 | 10.2301391 | -3.479993138 |
| C | -2.2432382 | 9.62953463 | -4.480363726 |
| H | -2.4306913 | 10.216847 | -5.383769236 |
| C | -1.0801832 | 8.88106944 | -4.402669625 |
| H | -0.3554981 | 8.90482707 | -5.223643288 |
| C | -0.8192671 | 8.10601477 | -3.246301643 |
| H | 1.20783228 | 3.50872236 | -4.75871309 |
| H | 0.27848164 | 5.66666864 | -6.52767122 |
| H | 3.10572018 | 6.2143233 | -6.668835003 |
| H | 5.76628695 | 5.55578137 | -5.088937222 |
| H | 6.72785592 | 4.16369439 | -4.690305121 |
| H | 0.10532509 | -1.0108504 | 1.306714201 |
| H | -3.7255696 | 1.66705053 | -2.958213782 |
| H | -3.1956775 | 1.34882962 | 6.270491007 |
| H | -4.0625038 | 2.6818033 | 5.245507668 |
| H | 0.06498256 | 12.6825431 | 6.387799786 |
| H | 1.39160431 | 11.8178918 | 7.361367736 |
| H | 0.80660913 | 7.54050594 | 8.333747778 |
| H | 0.89519107 | 6.0293407 | 7.23089412 |

Cartesian coordinates (in Å) for **wt QM region at 500 ps**:

| N | 0.59285772 | 3.76180836 | -5.67695182 |
| --- | --- | --- | --- |
| H | -0.0715523 | 4.18447529 | -5.03432041 |
| C | 1.19384869 | 4.63203869 | -6.698481073 |
| H | 1.55813116 | 4.01185729 | -7.539465331 |
| C | 2.28415611 | 5.63745586 | -6.258825397 |
| O | 2.17520461 | 6.21336699 | -5.179863067 |
| C | 6.57331903 | 5.0028005 | -4.657179636 |
| H | 7.48465861 | 5.53936285 | -4.975558695 |
| C | 6.40038407 | 5.23520168 | -3.145375287 |
| H | 6.16761684 | 6.30596364 | -3.002069736 |
| H | 7.36743037 | 5.10714312 | -2.622563017 |
| N | 5.26582727 | 4.37426069 | -1.067638649 |
| H | 5.93202218 | 4.83984778 | -0.429611117 |
| C | 5.371456 | 4.40317681 | -2.441644016 |
| C | 4.26946332 | 3.50059397 | -0.745750609 |
| H | 3.97357566 | 3.28344964 | 0.281178439 |
| N | 3.72369755 | 2.97208878 | -1.823384075 |
| C | 4.40515652 | 3.5252542 | -2.88696005 |
| H | 4.17786889 | 3.25445917 | -3.91790208 |
| C | 0.08447477 | -0.1233478 | 1.684806793 |
| H | -0.1097424 | -0.1579153 | 2.768937243 |
| C | 1.48269452 | 0.53817655 | 1.433903784 |
| H | 1.71100094 | 0.51494391 | 0.352752351 |
| H | 2.25908035 | -0.0494571 | 1.952420972 |
| N | 1.84293344 | 2.3591859 | 3.187175014 |
| C | 1.53158885 | 1.97225761 | 1.895152233 |
| C | 1.56692237 | 3.64906085 | 3.271886945 |
| H | 1.63949578 | 4.26500284 | 4.168381465 |
| N | 1.11572383 | 4.13766314 | 2.090651511 |
| H | 0.67832555 | 5.06089226 | 1.983031919 |
| C | 1.09349647 | 3.07914096 | 1.201421996 |
| H | 0.72670997 | 3.15385668 | 0.183279056 |
| C | -0.9745054 | 0.73250361 | 0.995514299 |
| O | -0.9277925 | 0.99908128 | -0.210248517 |
| N | -1.9541439 | 1.25335953 | 1.785981042 |
| H | -2.0425963 | 0.94562361 | 2.757873397 |
| C | -2.9209359 | 2.22505484 | 1.298914036 |
| H | -3.7176018 | 2.28047686 | 2.052739718 |
| C | -2.2550293 | 3.60006967 | 1.20049098 |
| H | -1.4308645 | 3.54531239 | 0.478357152 |
| H | -1.8296234 | 3.82315709 | 2.194149277 |
| O | -3.1953867 | 4.58201248 | 0.807291677 |
| H | -2.7532253 | 5.41879298 | 0.502851483 |
| C | -3.6079139 | 1.71504855 | 0.0260248 |
| O | -4.0606585 | 0.57274373 | -0.032462858 |
| N | -3.650445 | 2.57563763 | -1.028500968 |
| H | -3.4561956 | 3.55795317 | -0.812154053 |
| C | -4.28331 | 2.28404819 | -2.300609228 |
| H | -5.23741 | 1.75378288 | -2.119952217 |
| C | -4.5571273 | 3.58193702 | -3.088708956 |
| H | -4.9641508 | 3.33160712 | -4.083552616 |
| H | -3.6145368 | 4.1338516 | -3.2573995 |
| N | -5.0284176 | 5.67132853 | -1.79019326 |
| C | -5.492363 | 4.51268282 | -2.365585201 |
| C | -6.0584435 | 6.21884371 | -1.177779807 |
| H | -6.0324357 | 7.12083013 | -0.562309742 |
| N | -7.1840746 | 5.47769183 | -1.349660197 |
| H | -8.0897316 | 5.6047306 | -0.892321401 |
| C | -6.8418102 | 4.37588248 | -2.096425095 |
| H | -7.5780575 | 3.61239821 | -2.329501919 |
| C | -3.7252517 | 2.06708236 | 6.091293928 |
| H | -4.5521491 | 2.11157442 | 6.830481603 |
| C | -2.7055384 | 3.14855215 | 6.561865409 |
| H | -1.9616341 | 3.41222052 | 5.788906376 |
| H | -2.1246584 | 2.74897472 | 7.420569668 |
| C | -3.3604055 | 4.44274223 | 7.103444386 |
| O | -2.9366112 | 5.57286014 | 6.710945578 |
| O | -4.3010182 | 4.30812154 | 7.919542384 |
| C | 0.61681787 | 11.4271196 | 5.99818083 |
| H | -0.115064 | 10.6685355 | 6.326850287 |
| C | 1.32878163 | 10.9301749 | 4.711504089 |
| H | 1.99279124 | 10.0651325 | 4.925136439 |
| H | 2.00986349 | 11.7081499 | 4.309684745 |
| C | 0.41156187 | 10.5054626 | 3.598661685 |
| C | -0.9704023 | 10.2229157 | 3.692313373 |
| H | -1.6174357 | 10.2598048 | 4.567806162 |
| N | -1.472288 | 9.9579162 | 2.469594307 |
| H | -2.4809877 | 9.84544578 | 2.287727676 |
| C | -0.458049 | 10.0270692 | 1.520756371 |
| C | 0.73519144 | 10.3916982 | 2.203980331 |
| C | 1.9213844 | 10.5706611 | 1.468415421 |
| H | 2.83861079 | 10.8860092 | 1.972491556 |
| C | 1.90296978 | 10.3469856 | 0.091003165 |
| H | 2.81696924 | 10.4713365 | -0.495941701 |
| C | -0.4858529 | 9.79380337 | 0.149743483 |
| H | -1.4078916 | 9.4985304 | -0.352491286 |
| C | 0.71520833 | 9.95382652 | -0.556853266 |
| H | 0.73004085 | 9.76647111 | -1.634468735 |
| C | 0.55365617 | 6.75312655 | 7.436117332 |
| H | -0.3885668 | 6.28773429 | 7.776447926 |
| C | 0.23587564 | 7.77986122 | 6.306214076 |
| H | 1.14332181 | 8.35832524 | 6.049513188 |
| H | -0.4914096 | 8.50981664 | 6.707858046 |
| N | -1.4534292 | 6.60967275 | 4.797259985 |
| H | -2.081081 | 6.18460613 | 5.554276046 |
| C | -0.292738 | 7.2906567 | 5.002432355 |
| C | -1.5043072 | 6.30405055 | 3.470565385 |
| H | -2.3462699 | 5.76956453 | 3.028703841 |
| N | -0.4328466 | 6.73879668 | 2.802894402 |
| C | 0.32326979 | 7.3585411 | 3.730153843 |
| H | 1.28979811 | 7.79625338 | 3.49793601 |
| N | 0.03487676 | 6.91635515 | -3.465603619 |
| H | 0.72394857 | 6.8807141 | -4.223984391 |
| C | 0.33867419 | 6.21146706 | -2.338468311 |
| C | 1.63851714 | 5.4654813 | -2.248203838 |
| H | 2.27855737 | 5.89624502 | -1.454405638 |
| H | 1.50028494 | 4.39661293 | -2.010298642 |
| H | 2.22345209 | 5.53062661 | -3.170277735 |
| C | -0.5327968 | 6.21427231 | -1.27330276 |
| O | -0.2416662 | 5.51943642 | -0.139176164 |
| H | 0.69466446 | 5.26352118 | -0.151025654 |
| C | -1.7635634 | 6.95994798 | -1.271026244 |
| O | -2.5232865 | 6.96204312 | -0.27790917 |
| C | -2.0172311 | 7.73043862 | -2.489672446 |
| C | -3.1705773 | 8.53998099 | -2.601242309 |
| H | -3.8623744 | 8.54462337 | -1.756211449 |
| C | -3.4072534 | 9.28532364 | -3.743407804 |
| H | -4.3053734 | 9.90611599 | -3.822005599 |
| C | -2.4949214 | 9.22598936 | -4.821314536 |
| H | -2.6807961 | 9.79001487 | -5.738536348 |
| C | -1.3545234 | 8.44280616 | -4.742840379 |
| H | -0.6446216 | 8.42667357 | -5.577059614 |
| C | -1.098368 | 7.68880155 | -3.57334552 |
| H | 1.15883869 | 3.06346171 | -5.238830845 |
| H | 0.37380858 | 5.23714895 | -7.085059814 |
| H | 3.10650328 | 5.85492105 | -6.940383422 |
| H | 5.76436299 | 5.32755672 | -5.311542575 |
| H | 6.77696394 | 3.95667129 | -4.885674125 |
| H | 0.04692299 | -1.1236713 | 1.253517951 |
| H | -3.7163152 | 1.58676747 | -2.917361918 |
| H | -3.3364243 | 1.05377188 | 6.191705266 |
| H | -4.2159767 | 2.38888878 | 5.172764184 |
| H | 0.06506796 | 12.3507595 | 5.823456095 |
| H | 1.33438021 | 11.4312573 | 6.81864076 |
| H | 0.98249684 | 7.36213592 | 8.231900337 |
| H | 1.12921497 | 5.92427123 | 7.024051513 |

Cartesian coordinates (in Å) for **D126A QM region at 300 ps**:

| N | 0.78737919 | 4.48812025 | -4.968625213 |
| --- | --- | --- | --- |
| H | -0.1781388 | 4.4792613 | -4.663320209 |
| C | 1.13673046 | 5.20431374 | -6.178924597 |
| H | 1.43871995 | 4.53478756 | -7.007115462 |
| C | -0.1073075 | 6.00391268 | -6.648103944 |
| H | -0.5167428 | 6.53127966 | -5.767083932 |
| H | 0.26657701 | 6.7876581 | -7.327630558 |
| C | -1.1597818 | 5.20108113 | -7.346628853 |
| C | -2.3268744 | 4.66208298 | -6.840792274 |
| H | -2.7412101 | 4.7062708 | -5.832871754 |
| N | -3.0114713 | 3.99489838 | -7.828392523 |
| H | -3.9534359 | 3.59687587 | -7.714305909 |
| C | -2.3092995 | 4.08758649 | -9.007371429 |
| C | -1.1348946 | 4.85300544 | -8.745967796 |
| C | -0.2499217 | 5.14350695 | -9.803863644 |
| H | 0.65279427 | 5.73947447 | -9.631669014 |
| C | -0.5484788 | 4.66878442 | -11.07669269 |
| H | 0.13036982 | 4.90265167 | -11.90327688 |
| C | -2.584031 | 3.57396913 | -10.28297624 |
| H | -3.4500177 | 2.93024289 | -10.44939768 |
| C | -1.7019539 | 3.88321475 | -11.31389916 |
| H | -1.9041673 | 3.50405943 | -12.32084387 |
| C | 2.26157239 | 6.23811502 | -5.954580115 |
| O | 2.23981607 | 7.02708494 | -5.003379036 |
| N | 3.23822542 | 6.26555343 | -6.876331935 |
| H | 3.22406627 | 5.59272805 | -7.659144363 |
| C | 4.24586473 | 7.31800624 | -6.907588323 |
| H | 4.80065919 | 7.14314621 | -7.843508711 |
| C | 3.7049209 | 8.76380464 | -6.944517461 |
| H | 3.46860528 | 9.12650769 | -5.936642411 |
| H | 4.4859906 | 9.43001117 | -7.338962792 |
| S | 2.15509759 | 8.98687939 | -7.871638014 |
| H | 2.60617795 | 8.5954392 | -9.094316657 |
| C | 5.2927321 | 7.17753513 | -5.778572599 |
| O | 5.84957849 | 8.15827615 | -5.301047382 |
| N | 5.59546142 | 5.90754161 | -5.412008465 |
| H | 5.07824696 | 5.12453639 | -5.802876657 |
| C | 6.76284256 | 5.61561824 | -4.593650388 |
| H | 7.60084813 | 6.22795234 | -4.965424498 |
| C | 6.62750668 | 5.89247533 | -3.091015646 |
| H | 7.63627024 | 5.89266209 | -2.637211545 |
| H | 6.27723596 | 6.93616772 | -3.016855616 |
| N | 5.72304279 | 4.9808502 | -0.933037553 |
| H | 6.4239 | 5.47295678 | -0.360303928 |
| C | 5.72264302 | 4.99068035 | -2.310237607 |
| C | 4.79799286 | 4.06400777 | -0.527699273 |
| H | 4.57987506 | 3.85547102 | 0.520217381 |
| N | 4.20436291 | 3.48989518 | -1.553220512 |
| C | 4.77298506 | 4.05848004 | -2.671587779 |
| H | 4.47497644 | 3.76749997 | -3.67799668 |
| C | 0.43076118 | 0.45553672 | 1.695901348 |
| H | 0.25160173 | 0.38450696 | 2.781576168 |
| C | 1.83326439 | 1.09226561 | 1.474213963 |
| H | 2.60317269 | 0.37706983 | 1.814554606 |
| H | 1.99696672 | 1.27970658 | 0.398451209 |
| N | 2.20814422 | 2.37809887 | 3.606316913 |
| C | 1.96469921 | 2.37614215 | 2.244152443 |
| C | 2.05209354 | 3.62832624 | 4.009430766 |
| H | 2.12398515 | 4.01018379 | 5.028226119 |
| N | 1.7270783 | 4.43524247 | 2.971355244 |
| H | 1.51597307 | 5.42928024 | 3.032403459 |
| C | 1.67575164 | 3.66153326 | 1.834004349 |
| H | 1.40855328 | 4.08219931 | 0.86886457 |
| C | -0.5894261 | 1.38728934 | 1.050183748 |
| O | -0.571707 | 1.63568202 | -0.160216608 |
| N | -1.4928764 | 1.96995099 | 1.879293715 |
| H | -1.5098073 | 1.70785093 | 2.869995843 |
| C | -2.4442335 | 2.9703278 | 1.431003297 |
| H | -3.2190267 | 3.04212172 | 2.207931455 |
| C | -1.7784156 | 4.3410214 | 1.327567254 |
| H | -1.2534274 | 4.52690257 | 2.27691062 |
| H | -1.0262631 | 4.33158616 | 0.521207263 |
| O | -2.7827728 | 5.31190219 | 1.083624 |
| H | -2.3896545 | 6.19704982 | 0.841649258 |
| C | -3.1615085 | 2.47139496 | 0.170150776 |
| O | -3.6164954 | 1.329891 | 0.12041872 |
| N | -3.2097848 | 3.33973309 | -0.874962199 |
| H | -3.0447417 | 4.31825179 | -0.62607228 |
| C | -3.8706277 | 3.0964317 | -2.146164349 |
| H | -4.8367874 | 2.58961954 | -1.964651069 |
| C | -4.1434897 | 4.43973875 | -2.861883007 |
| H | -3.1911173 | 4.97241722 | -3.044632668 |
| H | -4.6045358 | 4.25112825 | -3.847987372 |
| N | -6.3960143 | 5.00852495 | -1.90451623 |
| C | -5.0580858 | 5.30911775 | -2.047955192 |
| C | -6.8702489 | 5.87870508 | -1.033431361 |
| H | -7.8936985 | 5.91802057 | -0.665541347 |
| N | -5.9043502 | 6.73960793 | -0.616297693 |
| H | -5.9726443 | 7.48424639 | 0.0934266 |
| C | -4.7398681 | 6.39137385 | -1.255410662 |
| H | -3.8093859 | 6.91511274 | -1.051714357 |
| C | -2.6627457 | 2.70031973 | 6.289677097 |
| H | -3.2350833 | 3.00303483 | 7.192606521 |
| C | -1.2808969 | 3.38058273 | 6.330580072 |
| H | -1.3093084 | 4.48128269 | 6.318611401 |
| H | -0.7416041 | 3.08771725 | 7.252815923 |
| H | -0.6388102 | 3.08004332 | 5.480446493 |
| C | 1.76603778 | 11.6912868 | 5.14935904 |
| H | 2.50329157 | 12.4563757 | 4.840553035 |
| H | 2.34401678 | 10.7897943 | 5.433715162 |
| C | 0.81069495 | 11.3966358 | 4.013083564 |
| C | -0.5574595 | 11.1966225 | 4.023301935 |
| H | -1.2561703 | 11.2091732 | 4.861722057 |
| N | -1.0243653 | 10.9809246 | 2.746752846 |
| H | -1.9402873 | 10.5824618 | 2.539813863 |
| C | 0.04037963 | 10.9763222 | 1.874405429 |
| C | 1.21131491 | 11.2549982 | 2.635184442 |
| C | 2.44793718 | 11.3217172 | 1.971165057 |
| H | 3.35265974 | 11.5637218 | 2.535474233 |
| C | 2.50105451 | 11.0876867 | 0.598222259 |
| H | 3.45984725 | 11.1223125 | 0.073125333 |
| C | 0.08453083 | 10.7585356 | 0.491368675 |
| H | -0.8275434 | 10.5594175 | -0.075625599 |
| C | 1.32776453 | 10.8114979 | -0.136657385 |
| H | 1.39903694 | 10.6360067 | -1.214678644 |
| C | 0.76322391 | 7.58958031 | 7.565776453 |
| H | -0.2330885 | 7.17476475 | 7.816127946 |
| C | 0.57792763 | 8.4603675 | 6.282797061 |
| H | -0.0660088 | 9.32784004 | 6.52961815 |
| H | 1.55043515 | 8.87216362 | 5.9585627 |
| N | -1.1672178 | 7.0283341 | 5.137124267 |
| H | -1.6831979 | 6.76130567 | 5.991538646 |
| C | -0.0161749 | 7.77877574 | 5.091350779 |
| C | -1.4501779 | 6.58301238 | 3.890283362 |
| H | -2.2861874 | 5.9099939 | 3.684985121 |
| N | -0.5491697 | 7.03532 | 3.022790998 |
| C | 0.34670044 | 7.78086613 | 3.760974004 |
| H | 1.1700927 | 8.30957731 | 3.285240346 |
| C | 1.66173316 | 6.38177297 | 7.231944008 |
| O | 1.20450297 | 5.53997813 | 6.468201378 |
| N | 0.50498384 | 7.67990987 | -2.887029215 |
| H | 1.11932534 | 7.525023 | -3.701329021 |
| C | 0.83576232 | 7.04931208 | -1.729747926 |
| C | 2.12890472 | 6.29725727 | -1.683830057 |
| H | 2.91720033 | 6.93285694 | -1.238848852 |
| H | 2.04135772 | 5.38827618 | -1.070659469 |
| H | 2.46656215 | 6.02475645 | -2.693907589 |
| C | -0.0097853 | 7.12660965 | -0.62705659 |
| O | 0.35398225 | 6.49921931 | 0.512994752 |
| H | -0.1624334 | 6.79118156 | 1.337129014 |
| C | -1.2677571 | 7.80129167 | -0.728549951 |
| O | -2.1324098 | 7.73254332 | 0.202140802 |
| C | -1.5155982 | 8.55951474 | -1.941085057 |
| C | -2.653061 | 9.39604078 | -2.065805404 |
| H | -3.3347409 | 9.46107666 | -1.213655706 |
| C | -2.877752 | 10.1102751 | -3.227684811 |
| H | -3.7532726 | 10.7612462 | -3.314763418 |
| C | -1.978763 | 9.99283417 | -4.313825227 |
| H | -2.1633446 | 10.5341316 | -5.244302443 |
| C | -0.8572284 | 9.18650292 | -4.222023343 |
| H | -0.1592371 | 9.11688893 | -5.061772045 |
| C | -0.6054257 | 8.47099297 | -3.025818384 |
| H | 1.35434864 | 3.7304705 | -4.645383807 |
| H | 7.00326035 | 4.57882392 | -4.828861211 |
| H | 0.34498733 | -0.5168154 | 1.210884407 |
| H | -3.3210198 | 2.41862439 | -2.799291944 |
| H | -2.5106469 | 1.6240173 | 6.370338919 |
| H | -3.2679659 | 3.09141892 | 5.471864568 |
| H | 1.26451058 | 12.0936547 | 6.029494188 |
| H | 1.05830511 | 8.25405185 | 8.377855251 |
| H | 2.67587721 | 6.25161708 | 7.609619273 |

Cartesian coordinates (in Å) for **D126A QM region at 400 ps**:

| N | 1.44067784 | 4.01804089 | -5.022722826 |
| --- | --- | --- | --- |
| H | 1.17777849 | 3.1424474 | -5.495577762 |
| C | 1.37874628 | 5.21208349 | -5.856851621 |
| H | 1.57190983 | 4.80508007 | -6.859050481 |
| C | 0.01079197 | 5.97311587 | -5.968142094 |
| H | -0.3773235 | 6.156072 | -4.951589361 |
| H | 0.2115746 | 6.96672613 | -6.406800296 |
| C | -0.9770506 | 5.22815965 | -6.811830882 |
| C | -1.9675036 | 4.36183612 | -6.39850034 |
| H | -2.2726941 | 4.08869912 | -5.388682996 |
| N | -2.6084724 | 3.80700801 | -7.480762772 |
| H | -3.2669011 | 3.01628422 | -7.422071121 |
| C | -2.0267421 | 4.27080161 | -8.634628409 |
| C | -0.9951681 | 5.1885121 | -8.257342793 |
| C | -0.2312696 | 5.81359045 | -9.271375863 |
| H | 0.57131722 | 6.51479117 | -9.019691131 |
| C | -0.5098976 | 5.52115585 | -10.60550726 |
| H | 0.0780914 | 6.00517299 | -11.39335633 |
| C | -2.2913249 | 3.96527621 | -9.977342285 |
| H | -3.0536547 | 3.2321616 | -10.24959557 |
| C | -1.5326082 | 4.60694701 | -10.95185242 |
| H | -1.7321024 | 4.380735 | -12.00352561 |
| C | 2.48567505 | 6.25652266 | -5.611221421 |
| O | 2.58130522 | 6.91677672 | -4.57563583 |
| N | 3.31120734 | 6.48119211 | -6.664864957 |
| H | 3.21925299 | 5.89092946 | -7.503412453 |
| C | 4.26692012 | 7.58671862 | -6.698632836 |
| H | 4.74288118 | 7.51652655 | -7.690587362 |
| C | 3.64909507 | 8.98486848 | -6.565072271 |
| H | 3.37943372 | 9.20624466 | -5.526511369 |
| H | 4.39038091 | 9.73027303 | -6.886287885 |
| S | 2.14927239 | 9.09879497 | -7.607227969 |
| H | 2.0074536 | 10.4449768 | -7.484773686 |
| C | 5.40494008 | 7.43727466 | -5.66712267 |
| O | 5.91839636 | 8.41389258 | -5.135835235 |
| N | 5.81927123 | 6.17031552 | -5.443658467 |
| H | 5.35064213 | 5.39265501 | -5.897022238 |
| C | 6.99804206 | 5.88219994 | -4.645915183 |
| H | 7.80304494 | 6.56652163 | -4.962588437 |
| C | 6.83396742 | 6.04782028 | -3.130717818 |
| H | 7.82811519 | 5.96522774 | -2.652900646 |
| H | 6.52858972 | 7.09857049 | -2.980795899 |
| N | 5.88284658 | 5.01674777 | -1.064354619 |
| H | 6.5745987 | 5.46696154 | -0.447954748 |
| C | 5.86957168 | 5.13500855 | -2.437490477 |
| C | 4.90458064 | 4.13279057 | -0.719310995 |
| H | 4.68830918 | 3.85414755 | 0.312431503 |
| N | 4.26270423 | 3.68011086 | -1.776247426 |
| C | 4.85070694 | 4.3001277 | -2.855454149 |
| H | 4.48473073 | 4.12473127 | -3.866781436 |
| C | 0.59680653 | 0.29839335 | 1.59033323 |
| H | 0.45570458 | 0.26051434 | 2.683571064 |
| C | 1.99068949 | 0.92343211 | 1.305638275 |
| H | 2.7738302 | 0.26588053 | 1.723993622 |
| H | 2.16005387 | 0.99113091 | 0.216646593 |
| N | 2.08777226 | 2.52294366 | 3.265169526 |
| C | 2.07980471 | 2.30213753 | 1.898466542 |
| C | 1.94734347 | 3.82801421 | 3.42973533 |
| H | 1.89980988 | 4.35552988 | 4.385494115 |
| N | 1.84917388 | 4.46289334 | 2.233104899 |
| H | 1.64558743 | 5.44727403 | 2.059745602 |
| C | 1.93317177 | 3.50928683 | 1.247174947 |
| H | 1.8621296 | 3.75381404 | 0.190064752 |
| C | -0.4508975 | 1.2099228 | 0.962111224 |
| O | -0.4907116 | 1.43724494 | -0.249256104 |
| N | -1.3226343 | 1.8002432 | 1.820610532 |
| H | -1.2978127 | 1.55414157 | 2.814140034 |
| C | -2.2940715 | 2.79178556 | 1.405017335 |
| H | -3.0517301 | 2.84411433 | 2.202173135 |
| C | -1.64472 | 4.17300316 | 1.313705359 |
| H | -1.1261979 | 4.36456236 | 2.269219055 |
| H | -0.8833559 | 4.17932496 | 0.513203336 |
| O | -2.6599448 | 5.12108749 | 1.071514211 |
| H | -2.2969599 | 6.01941481 | 0.807265633 |
| C | -3.0513728 | 2.29445148 | 0.160228676 |
| O | -3.5183222 | 1.15704469 | 0.131274363 |
| N | -3.1364741 | 3.16057226 | -0.882727531 |
| H | -2.9290337 | 4.1330043 | -0.645995715 |
| C | -3.88387 | 2.93997513 | -2.115628563 |
| H | -4.8411976 | 2.44116041 | -1.876958787 |
| C | -4.1841855 | 4.30073035 | -2.782395645 |
| H | -3.2421171 | 4.85762785 | -2.940985469 |
| H | -4.6363878 | 4.13770408 | -3.775626995 |
| N | -6.4918211 | 4.847363 | -1.966767708 |
| C | -5.1410205 | 5.12121826 | -1.957607641 |
| C | -7.0428777 | 5.65518716 | -1.087411815 |
| H | -8.0989202 | 5.69388756 | -0.823765481 |
| N | -6.1087885 | 6.45894703 | -0.51260924 |
| H | -6.2594183 | 7.10455399 | 0.256542239 |
| C | -4.885624 | 6.13309908 | -1.054116702 |
| H | -3.9689195 | 6.62704791 | -0.733730322 |
| C | -2.5702474 | 2.60946687 | 6.277835664 |
| H | -3.133159 | 2.90690006 | 7.189250035 |
| C | -1.2071729 | 3.33025785 | 6.302196122 |
| H | -1.2673468 | 4.42908218 | 6.311024935 |
| H | -0.642432 | 3.03871786 | 7.209539067 |
| H | -0.5740623 | 3.05723091 | 5.436795677 |
| C | 1.6933282 | 11.7567034 | 5.32966066 |
| H | 2.32416681 | 12.6195783 | 5.04230861 |
| H | 2.39133195 | 10.930768 | 5.5752172 |
| C | 0.79762611 | 11.3535806 | 4.18490335 |
| C | -0.5524546 | 11.0621599 | 4.139514607 |
| H | -1.2966132 | 11.0634793 | 4.938754627 |
| N | -0.9297631 | 10.7295883 | 2.854083503 |
| H | -1.7917673 | 10.2241618 | 2.62860464 |
| C | 0.19123937 | 10.7281936 | 2.049352896 |
| C | 1.29288406 | 11.1414116 | 2.848084766 |
| C | 2.56726741 | 11.2299855 | 2.258634286 |
| H | 3.42822569 | 11.5641507 | 2.846816414 |
| C | 2.71945436 | 10.8824015 | 0.917612814 |
| H | 3.70449318 | 10.9377251 | 0.446667877 |
| C | 0.33738358 | 10.3993392 | 0.697622053 |
| H | -0.5259791 | 10.1120435 | 0.096548579 |
| C | 1.61271266 | 10.4794015 | 0.141382937 |
| H | 1.76226114 | 10.2259182 | -0.912268404 |
| C | 0.83319896 | 7.65001686 | 7.666530783 |
| H | -0.1608166 | 7.26903439 | 7.972460236 |
| C | 0.58788354 | 8.49289904 | 6.373719088 |
| H | -0.0790529 | 9.33680111 | 6.636152754 |
| H | 1.53677557 | 8.93209842 | 6.015644594 |
| N | -1.1846498 | 7.05797821 | 5.257555711 |
| H | -1.7248497 | 6.82767162 | 6.109233156 |
| C | -0.0192801 | 7.78516858 | 5.202390654 |
| C | -1.4769863 | 6.60756588 | 4.016222474 |
| H | -2.3186545 | 5.94170139 | 3.810845571 |
| N | -0.5663046 | 7.02996671 | 3.147302826 |
| C | 0.34723887 | 7.75836708 | 3.874125874 |
| H | 1.19384226 | 8.24602379 | 3.395555531 |
| C | 1.66659831 | 6.40676823 | 7.304326768 |
| O | 1.09716984 | 5.50260357 | 6.707173147 |
| N | 0.7632746 | 7.95390275 | -2.568755088 |
| H | 1.44004658 | 7.83634481 | -3.330437437 |
| C | 1.06616483 | 7.34757011 | -1.393198187 |
| C | 2.46255183 | 6.85608894 | -1.186411498 |
| H | 3.08656509 | 7.66986016 | -0.770443748 |
| H | 2.47131242 | 6.00893877 | -0.489115569 |
| H | 2.9115533 | 6.53850027 | -2.137822337 |
| C | 0.09058883 | 7.21700442 | -0.410480296 |
| O | 0.41229146 | 6.59131955 | 0.746274872 |
| H | -0.1770352 | 6.82626331 | 1.548015349 |
| C | -1.2311764 | 7.70000837 | -0.636860786 |
| O | -2.1709624 | 7.49329423 | 0.203078984 |
| C | -1.4634247 | 8.46528898 | -1.845454474 |
| C | -2.6862291 | 9.14862045 | -2.059815396 |
| H | -3.4429278 | 9.1002073 | -1.27363834 |
| C | -2.8998369 | 9.86334292 | -3.223086785 |
| H | -3.8408275 | 10.4010466 | -3.377023189 |
| C | -1.9050226 | 9.8819335 | -4.228491301 |
| H | -2.0800704 | 10.4209548 | -5.162241706 |
| C | -0.6992198 | 9.22381918 | -4.047900687 |
| H | 0.06610485 | 9.25472914 | -4.830128215 |
| C | -0.4484065 | 8.54095535 | -2.833125536 |
| H | 1.19521837 | 3.98574226 | -4.053868378 |
| H | 7.29214516 | 4.88280608 | -4.966512447 |
| H | 0.49428249 | -0.6860454 | 1.133784755 |
| H | -3.3927275 | 2.25151383 | -2.803282092 |
| H | -2.3824821 | 1.53916891 | 6.363122347 |
| H | -3.1965186 | 3.00096068 | 5.476222166 |
| H | 1.15635688 | 12.0606762 | 6.22817592 |
| H | 1.19409089 | 8.31545937 | 8.450756897 |
| H | 2.72875154 | 6.31221439 | 7.530064229 |

Cartesian coordinates (in Å) for **D126A QM region at 500 ps**:

| N | 1.43876031 | 4.683549 | -4.67119584 |
| --- | --- | --- | --- |
| H | 1.60484634 | 5.18565489 | -3.806406401 |
| C | 1.45759644 | 5.43899758 | -5.911606762 |
| H | 1.73013244 | 4.78886841 | -6.759867329 |
| C | 0.13029011 | 6.20084592 | -6.256006142 |
| H | -0.2934912 | 6.56346408 | -5.3048744 |
| H | 0.39656509 | 7.09905814 | -6.842512542 |
| C | -0.8748602 | 5.40010651 | -7.016689421 |
| C | -2.0157205 | 4.79571878 | -6.537959619 |
| H | -2.4347544 | 4.78854472 | -5.531713544 |
| N | -2.6654358 | 4.12814516 | -7.555984193 |
| H | -3.580001 | 3.68301504 | -7.424856072 |
| C | -1.9628103 | 4.30311519 | -8.733522536 |
| C | -0.8256242 | 5.10741957 | -8.430475917 |
| C | 0.06929154 | 5.45796642 | -9.462050213 |
| H | 0.94457635 | 6.07991681 | -9.254096424 |
| C | -0.1782731 | 4.9995808 | -10.75198985 |
| H | 0.50824984 | 5.27177234 | -11.56186874 |
| C | -2.1949003 | 3.81532706 | -10.02727151 |
| H | -3.0335221 | 3.15440924 | -10.249644 |
| C | -1.2969578 | 4.17900464 | -11.02589848 |
| H | -1.4708661 | 3.81446916 | -12.04252173 |
| C | 2.51812658 | 6.522944 | -5.755093654 |
| O | 2.58839457 | 7.18081244 | -4.709601739 |
| N | 3.30097972 | 6.77297915 | -6.819839166 |
| H | 3.28023571 | 6.1299117 | -7.630335651 |
| C | 4.28049571 | 7.85854116 | -6.825029361 |
| H | 4.79435622 | 7.76556818 | -7.79383166 |
| C | 3.6921462 | 9.27576309 | -6.726599102 |
| H | 3.49840262 | 9.55706836 | -5.686531927 |
| H | 4.42417445 | 9.98915162 | -7.132257127 |
| S | 2.11979763 | 9.39668472 | -7.664201643 |
| H | 2.03588933 | 10.7520841 | -7.597912414 |
| C | 5.35667675 | 7.66087199 | -5.731206581 |
| O | 5.88035144 | 8.61024917 | -5.165366345 |
| N | 5.68746048 | 6.36946553 | -5.483523971 |
| H | 5.20148328 | 5.62088178 | -5.966062142 |
| C | 6.7894003 | 6.00165114 | -4.612534199 |
| H | 7.68181068 | 6.577096 | -4.913747053 |
| C | 6.56106107 | 6.24449187 | -3.11421408 |
| H | 7.54092631 | 6.19788747 | -2.605756009 |
| H | 6.24280185 | 7.29759264 | -3.022826922 |
| N | 5.63453502 | 5.18942487 | -1.042615713 |
| H | 6.31254373 | 5.67911982 | -0.433343293 |
| C | 5.59911843 | 5.33291272 | -2.410296204 |
| C | 4.70253465 | 4.26230221 | -0.69391193 |
| H | 4.527435 | 3.94846781 | 0.336294459 |
| N | 4.06120197 | 3.80557097 | -1.751454202 |
| C | 4.60783059 | 4.46544905 | -2.828535593 |
| H | 4.24355274 | 4.29157307 | -3.840733507 |
| C | 0.55013093 | 0.35824904 | 1.662166479 |
| H | 0.37137441 | 0.32495556 | 2.749790372 |
| C | 1.9332913 | 1.0297791 | 1.428934373 |
| H | 2.7207063 | 0.39396267 | 1.873007811 |
| H | 2.13857583 | 1.11283316 | 0.346987162 |
| N | 1.94899236 | 2.58146216 | 3.416015174 |
| C | 1.96242067 | 2.39733452 | 2.047059413 |
| C | 1.77588517 | 3.876986 | 3.613529912 |
| H | 1.70561246 | 4.38306539 | 4.579890828 |
| N | 1.67533017 | 4.54028924 | 2.432011471 |
| H | 1.49076414 | 5.53088102 | 2.296074471 |
| C | 1.7957907 | 3.61444311 | 1.421622111 |
| H | 1.73174737 | 3.88233777 | 0.370231585 |
| C | -0.5062262 | 1.23456495 | 0.991373122 |
| O | -0.5558891 | 1.40745258 | -0.228915286 |
| N | -1.3807172 | 1.85639285 | 1.830431202 |
| H | -1.3153871 | 1.68281812 | 2.837117062 |
| C | -2.3529173 | 2.82975684 | 1.37582021 |
| H | -3.0994764 | 2.92953409 | 2.177015985 |
| C | -1.7076038 | 4.20626833 | 1.198420824 |
| H | -1.1874332 | 4.4565942 | 2.137207416 |
| H | -0.9551769 | 4.16772095 | 0.3909743 |
| O | -2.7308369 | 5.14204475 | 0.909164373 |
| H | -2.3735048 | 6.05543211 | 0.714370996 |
| C | -3.1363742 | 2.26948285 | 0.178045192 |
| O | -3.5843337 | 1.1241928 | 0.204308566 |
| N | -3.2789851 | 3.10031238 | -0.889193242 |
| H | -3.0909391 | 4.0870807 | -0.689169682 |
| C | -4.0751607 | 2.82077559 | -2.076028768 |
| H | -5.0246213 | 2.33903523 | -1.777984696 |
| C | -4.3916915 | 4.13694776 | -2.815964403 |
| H | -3.4512152 | 4.66445215 | -3.063600182 |
| H | -4.8977345 | 3.92641941 | -3.77408855 |
| N | -6.6069049 | 4.7878138 | -1.787647947 |
| C | -5.2685727 | 5.04303342 | -1.999423918 |
| C | -7.0383028 | 5.73720056 | -0.981446801 |
| H | -8.0519345 | 5.83651305 | -0.593002473 |
| N | -6.0412348 | 6.60828879 | -0.672846695 |
| H | -6.0482574 | 7.39710451 | -0.020037382 |
| C | -4.90367 | 6.17635422 | -1.308594649 |
| H | -3.9540112 | 6.68890934 | -1.183912089 |
| C | -2.7251929 | 2.60909156 | 6.313179293 |
| H | -3.4340142 | 2.77757477 | 7.153984061 |
| C | -1.5108287 | 3.53484316 | 6.528684663 |
| H | -1.7684475 | 4.60954018 | 6.569908905 |
| H | -1.0139525 | 3.28977674 | 7.488478317 |
| H | -0.7513305 | 3.42337587 | 5.734210887 |
| C | 1.65974666 | 11.912578 | 4.783877203 |
| H | 2.30925065 | 12.7385418 | 4.437237998 |
| H | 2.33198342 | 11.0865808 | 5.094091882 |
| C | 0.73764732 | 11.4746844 | 3.67193004 |
| C | -0.5956723 | 11.1164004 | 3.704165138 |
| H | -1.2826887 | 11.0694725 | 4.549834617 |
| N | -1.0460283 | 10.8082532 | 2.441645626 |
| H | -1.9616567 | 10.3987301 | 2.263106804 |
| C | -0.002879 | 10.9274701 | 1.552860942 |
| C | 1.13540975 | 11.3598662 | 2.292656087 |
| C | 2.34015378 | 11.6023563 | 1.608948811 |
| H | 3.20906879 | 11.978886 | 2.155589709 |
| C | 2.40198677 | 11.3773901 | 0.236238805 |
| H | 3.33375619 | 11.555657 | -0.306324104 |
| C | 0.0507597 | 10.7180776 | 0.170000014 |
| H | -0.8387794 | 10.419342 | -0.385841975 |
| C | 1.26569859 | 10.935076 | -0.476158807 |
| H | 1.33904106 | 10.7591851 | -1.552505847 |
| C | 0.78270092 | 7.90936864 | 7.398728255 |
| H | -0.1983778 | 7.54209059 | 7.758945073 |
| C | 0.4951436 | 8.71697392 | 6.091666056 |
| H | -0.1414138 | 9.58466593 | 6.350543329 |
| H | 1.43504983 | 9.12735144 | 5.681649726 |
| N | -1.3892201 | 7.36004182 | 5.040973629 |
| H | -2.0015942 | 7.30912854 | 5.869096501 |
| C | -0.1574754 | 7.97673202 | 4.969669194 |
| C | -1.6690469 | 6.81521121 | 3.824413816 |
| H | -2.5490353 | 6.2013142 | 3.622480931 |
| N | -0.6927606 | 7.07382815 | 2.972132069 |
| C | 0.24910207 | 7.78939635 | 3.667339957 |
| H | 1.14905816 | 8.16676982 | 3.185630386 |
| C | 1.60195533 | 6.65136253 | 7.050653346 |
| O | 0.99803161 | 5.71576086 | 6.537977428 |
| N | 0.62389889 | 8.01898312 | -2.724383168 |
| H | 1.3401967 | 8.03001114 | -3.457262505 |
| C | 0.94508325 | 7.42219604 | -1.544472993 |
| C | 2.36467189 | 7.00282664 | -1.316683702 |
| H | 2.87647455 | 7.7801612 | -0.718791548 |
| H | 2.4134348 | 6.05731299 | -0.757422289 |
| H | 2.92105844 | 6.9032313 | -2.259388862 |
| C | -0.0220897 | 7.27237896 | -0.559746665 |
| O | 0.31491767 | 6.64216464 | 0.586383 |
| H | -0.290412 | 6.85940171 | 1.38114126 |
| C | -1.3491943 | 7.78397611 | -0.745488224 |
| O | -2.2382014 | 7.62088261 | 0.14897734 |
| C | -1.6090982 | 8.50344926 | -1.981774792 |
| C | -2.8455339 | 9.15788 | -2.212552225 |
| H | -3.6003326 | 9.1270925 | -1.423031648 |
| C | -3.0821056 | 9.8251974 | -3.400031505 |
| H | -4.0401855 | 10.3266389 | -3.568679763 |
| C | -2.0911291 | 9.84427036 | -4.409789692 |
| H | -2.2822743 | 10.3413231 | -5.363970271 |
| C | -0.8646056 | 9.23574159 | -4.207664938 |
| H | -0.094398 | 9.27708576 | -4.984911536 |
| C | -0.601917 | 8.58028035 | -2.980265141 |
| H | 1.13071094 | 3.73405957 | -4.611631509 |
| H | 6.9887306 | 4.95820565 | -4.856575719 |
| H | 0.49232197 | -0.6342109 | 1.215233819 |
| H | -3.6102931 | 2.10023931 | -2.748927639 |
| H | -2.4151818 | 1.56834223 | 6.406994149 |
| H | -3.2853108 | 2.92069292 | 5.431567168 |
| H | 1.12428384 | 12.2986459 | 5.6512286 |
| H | 1.17816538 | 8.60793016 | 8.136079586 |
| H | 2.67578081 | 6.57856252 | 7.222892493 |

Cartesian coordinates (in Å) for **H251A QM region at 300 ps**:

| N | -0.9550774 | 5.58659286 | 4.584315558 |
| --- | --- | --- | --- |
| H | -1.1620679 | 6.14074508 | 3.76178094 |
| C | -1.1313389 | 6.24067386 | 5.872272515 |
| H | -1.5273281 | 5.54378736 | 6.630693455 |
| C | 0.1511916 | 6.9219006 | 6.43679431 |
| H | -0.1889424 | 7.65424992 | 7.193037176 |
| H | 0.6006338 | 7.50227358 | 5.61310353 |
| C | 1.17063037 | 6.02047187 | 7.060049542 |
| C | 2.40238458 | 5.65430248 | 6.555747422 |
| H | 2.88111362 | 5.92695273 | 5.616909633 |
| N | 3.06743254 | 4.84832522 | 7.457665652 |
| H | 4.0008539 | 4.4709232 | 7.285330099 |
| C | 2.27895189 | 4.67569638 | 8.578789578 |
| C | 1.07712209 | 5.41193529 | 8.366562317 |
| C | 0.09505172 | 5.43417708 | 9.377275899 |
| H | -0.8242671 | 6.01384228 | 9.246429561 |
| C | 0.31559124 | 4.7104272 | 10.54424115 |
| H | -0.4408316 | 4.72446211 | 11.33613855 |
| C | 2.49205433 | 3.93297411 | 9.749218188 |
| H | 3.39275612 | 3.33211131 | 9.885759631 |
| C | 1.4989567 | 3.95541815 | 10.72344215 |
| H | 1.64182612 | 3.37355807 | 11.63964044 |
| C | -2.1487044 | 7.3625524 | 5.637314656 |
| O | -2.0397325 | 8.10110936 | 4.651974224 |
| N | -3.1161942 | 7.4981647 | 6.563493313 |
| H | -3.1513309 | 6.84485588 | 7.361610171 |
| C | -4.1221115 | 8.55814494 | 6.542467025 |
| H | -4.6203763 | 8.49489163 | 7.524030595 |
| C | -3.5500578 | 9.97196845 | 6.39772872 |
| H | -4.331452 | 10.6944752 | 6.670522559 |
| H | -3.2390688 | 10.1659058 | 5.363204435 |
| S | -2.1133142 | 10.1393176 | 7.520756154 |
| H | -2.0636876 | 11.4973394 | 7.482402279 |
| C | -5.2311405 | 8.33436191 | 5.498890831 |
| O | -5.9811116 | 9.24477345 | 5.191020519 |
| N | -5.3426066 | 7.07205436 | 5.007862788 |
| H | -4.7313102 | 6.3363392 | 5.345085299 |
| C | -6.4785302 | 6.69279687 | 4.195567581 |
| H | -7.3357693 | 7.30070126 | 4.532118531 |
| C | -6.330969 | 6.89832108 | 2.674183439 |
| H | -5.9944031 | 7.93545512 | 2.532595543 |
| H | -7.336529 | 6.8615982 | 2.213233416 |
| N | -4.3057245 | 6.52544395 | 1.227290979 |
| C | -5.401737 | 6.01514993 | 1.885287671 |
| C | -3.8054212 | 5.52867274 | 0.526391183 |
| H | -2.9615624 | 5.57370724 | -0.165658552 |
| N | -4.4969881 | 4.37481797 | 0.721440375 |
| H | -4.4219237 | 3.54438709 | 0.096281758 |
| C | -5.5381303 | 4.67469031 | 1.572242414 |
| H | -6.2951705 | 3.94214175 | 1.845676091 |
| C | 0.1780505 | 1.70504326 | -1.938028631 |
| H | 0.35835002 | 1.67205242 | -3.024075222 |
| C | -1.2126161 | 2.35328631 | -1.694751519 |
| H | -1.3650885 | 2.50723107 | -0.612477217 |
| H | -1.9954626 | 1.65633094 | -2.034300347 |
| N | -1.727676 | 3.6938681 | -3.769056185 |
| C | -1.3389307 | 3.65266976 | -2.443459023 |
| C | -1.4853347 | 4.92453437 | -4.187307896 |
| H | -1.6290076 | 5.29725806 | -5.201701308 |
| N | -0.9820933 | 5.69913807 | -3.19994155 |
| H | -0.5057677 | 6.60108191 | -3.367497031 |
| C | -0.88291 | 4.90371654 | -2.076794543 |
| H | -0.4553051 | 5.26810348 | -1.148141319 |
| C | 1.20825823 | 2.61280671 | -1.286252782 |
| O | 1.24019754 | 2.82733526 | -0.069043887 |
| N | 2.05841965 | 3.26204257 | -2.130749934 |
| H | 2.09313091 | 2.97836385 | -3.113097026 |
| C | 3.01634335 | 4.23837328 | -1.655103081 |
| H | 3.73849181 | 4.4158107 | -2.463089419 |
| C | 2.34456132 | 5.58473232 | -1.358175771 |
| H | 1.60421156 | 5.45233543 | -0.551252129 |
| H | 1.811577 | 5.90982971 | -2.267811745 |
| O | 3.33896727 | 6.52127607 | -0.985457018 |
| H | 2.95718805 | 7.43211248 | -0.904928519 |
| C | 3.83964591 | 3.61656713 | -0.519584471 |
| O | 4.28160944 | 2.47477835 | -0.616034386 |
| N | 4.02480291 | 4.37252204 | 0.59741955 |
| H | 3.81347497 | 5.37010159 | 0.499158078 |
| C | 4.85408731 | 3.95890764 | 1.712923809 |
| H | 5.74995106 | 3.43996349 | 1.325367999 |
| C | 5.27436532 | 5.18035253 | 2.557759729 |
| H | 5.93054946 | 4.86474266 | 3.387620824 |
| H | 4.39131778 | 5.63849054 | 3.034620614 |
| N | 7.2074058 | 6.1728596 | 1.196554907 |
| H | 7.86759457 | 5.40559711 | 1.274649756 |
| C | 5.93607822 | 6.2445781 | 1.741229693 |
| C | 7.40452424 | 7.29380869 | 0.445727902 |
| H | 8.33396892 | 7.47860586 | -0.095781301 |
| N | 6.33862618 | 8.07306861 | 0.471520813 |
| C | 5.42959931 | 7.43603408 | 1.277051878 |
| H | 4.44588815 | 7.85827991 | 1.474750959 |
| C | 3.46915261 | 3.77563204 | -6.249288341 |
| H | 3.9904765 | 4.15725224 | -7.150961043 |
| C | 2.17044633 | 4.63996497 | -6.083937795 |
| H | 2.41989953 | 5.7123403 | -5.964553308 |
| H | 1.59925488 | 4.32902296 | -5.189828229 |
| C | 1.23100339 | 4.56913818 | -7.304023924 |
| O | 0.13367889 | 3.95928656 | -7.183970551 |
| O | 1.63512456 | 5.07786725 | -8.379769379 |
| C | -0.4921838 | 14.3590669 | -5.506475184 |
| H | 0.307057 | 13.7615662 | -5.981485326 |
| C | -1.0253515 | 13.5443472 | -4.300844015 |
| H | -1.4399157 | 12.5699527 | -4.641614668 |
| H | -1.87348 | 14.0584739 | -3.805673761 |
| C | -0.0171102 | 13.2256958 | -3.232748351 |
| C | 1.4054288 | 13.1976423 | -3.336082527 |
| H | 2.02516262 | 13.3563163 | -4.218249968 |
| N | 1.94929146 | 12.9353611 | -2.137465551 |
| H | 2.95547176 | 12.8810699 | -1.913173807 |
| C | 0.93129605 | 12.7555644 | -1.194129582 |
| C | -0.3076303 | 12.9456789 | -1.863320427 |
| C | -1.5097091 | 12.8707767 | -1.131290968 |
| H | -2.4736502 | 13.031061 | -1.622994595 |
| C | -1.4465413 | 12.5946786 | 0.236205013 |
| H | -2.3654346 | 12.5625824 | 0.825971132 |
| C | 1.00599096 | 12.4690401 | 0.157451853 |
| H | 1.96328922 | 12.337925 | 0.664724937 |
| C | -0.2100011 | 12.3968551 | 0.868653415 |
| H | -0.1778591 | 12.2083495 | 1.94360669 |
| C | -0.5561096 | 9.87253841 | -7.879888866 |
| H | 0.05660659 | 9.27800853 | -8.582511414 |
| C | 0.40190739 | 10.5577648 | -6.883885881 |
| H | 1.05718418 | 11.2694095 | -7.414759052 |
| H | 1.07558754 | 9.84434681 | -6.371543401 |
| H | -0.1304459 | 11.1349941 | -6.099761029 |
| N | 0.01874921 | 9.09307829 | 2.788636584 |
| H | -0.6208051 | 8.97372929 | 3.587054739 |
| C | -0.3683273 | 8.55153501 | 1.602464141 |
| C | -1.7279062 | 7.93451757 | 1.517123881 |
| H | -2.3092457 | 8.30649731 | 0.655893285 |
| H | -1.6563651 | 6.83912722 | 1.399611778 |
| H | -2.3340659 | 8.122142 | 2.412452961 |
| C | 0.4987935 | 8.54780255 | 0.522013506 |
| O | 0.18429583 | 7.97656815 | -0.674479959 |
| H | -0.7380515 | 7.67441149 | -0.689043254 |
| C | 1.8073978 | 9.12871944 | 0.60246028 |
| O | 2.60074057 | 9.08140099 | -0.382507169 |
| C | 2.15169848 | 9.74457364 | 1.875881825 |
| C | 3.38465886 | 10.4209577 | 2.049474977 |
| H | 4.0724068 | 10.4680632 | 1.202852344 |
| C | 3.70409434 | 11.0102967 | 3.258234354 |
| H | 4.6514379 | 11.5426352 | 3.371204323 |
| C | 2.80970411 | 10.913481 | 4.35004223 |
| H | 3.07209599 | 11.3384887 | 5.321691935 |
| C | 1.59049836 | 10.2715217 | 4.212915118 |
| H | 0.89543987 | 10.2234136 | 5.058126353 |
| C | 1.24175913 | 9.69689771 | 2.968256452 |
| O | 1.89127691 | 6.25262575 | 3.240768615 |
| O | 1.65399958 | 6.01857119 | 2.083721014 |
| H | -0.8652027 | 4.5971238 | 4.47097532 |
| H | -6.6942718 | 5.66306257 | 4.480471347 |
| H | 0.227119 | 0.70569716 | -1.505631839 |
| H | 4.38674932 | 3.20041185 | 2.340894076 |
| H | 3.20398145 | 2.73612487 | -6.442100199 |
| H | 4.16080235 | 4.03502242 | -5.447786875 |
| H | -0.0708363 | 15.3188033 | -5.207410578 |
| H | -1.2522819 | 14.3829908 | -6.287337445 |
| H | -1.1248553 | 10.6146308 | -8.440151864 |
| H | -1.1242002 | 9.11642171 | -7.338014393 |

Cartesian coordinates (in Å) for **H251A QM region at 400 ps**:

| N | -0.7063201 | 5.78595979 | 4.920243199 |
| --- | --- | --- | --- |
| H | 0.17178457 | 5.25091351 | 4.922610849 |
| C | -1.022569 | 6.56053279 | 6.125780449 |
| H | -1.5069964 | 5.94170882 | 6.90426363 |
| C | 0.27143788 | 7.15072217 | 6.734940017 |
| H | -0.0684885 | 7.90984841 | 7.463615411 |
| H | 0.79996716 | 7.68867364 | 5.929857324 |
| C | 1.19550895 | 6.19873837 | 7.425214506 |
| C | 2.43680982 | 5.77674748 | 6.990728976 |
| H | 2.96320289 | 6.02362851 | 6.06831712 |
| N | 2.9995915 | 4.92009499 | 7.904953524 |
| H | 3.76387371 | 4.28836234 | 7.668867697 |
| C | 2.12588271 | 4.74936635 | 8.96138435 |
| C | 0.98529468 | 5.56350787 | 8.704830741 |
| C | -0.0443936 | 5.62117247 | 9.66442092 |
| H | -0.9124959 | 6.26820408 | 9.507203734 |
| C | 0.06803322 | 4.8538632 | 10.82219806 |
| H | -0.7273913 | 4.89951519 | 11.57259768 |
| C | 2.24134943 | 3.9787953 | 10.12412141 |
| H | 3.1147677 | 3.34717365 | 10.30266197 |
| C | 1.19910587 | 4.03711099 | 11.04887195 |
| H | 1.27461936 | 3.44952897 | 11.9682824 |
| C | -1.938662 | 7.786359 | 5.850611554 |
| O | -1.6065631 | 8.69555939 | 5.083074444 |
| N | -3.1110936 | 7.74219614 | 6.504582807 |
| H | -3.3036983 | 6.90649535 | 7.078294648 |
| C | -4.2243177 | 8.66618241 | 6.328088251 |
| H | -4.8447788 | 8.53259536 | 7.227364001 |
| C | -3.8592383 | 10.150941 | 6.251462863 |
| H | -4.8032899 | 10.7111069 | 6.264735709 |
| H | -3.3163312 | 10.3780495 | 5.325011822 |
| S | -2.8383886 | 10.6259589 | 7.688767273 |
| H | -3.0078674 | 11.9709048 | 7.561211564 |
| C | -5.1802445 | 8.32685037 | 5.164806788 |
| O | -6.056348 | 9.13656489 | 4.896046409 |
| N | -5.0871588 | 7.11311063 | 4.553624427 |
| H | -4.2829449 | 6.45732986 | 4.649448522 |
| C | -6.1757789 | 6.73353697 | 3.673778496 |
| H | -7.0371243 | 7.34397678 | 3.990830478 |
| C | -5.9825024 | 7.00513795 | 2.171507728 |
| H | -5.6831013 | 8.0584103 | 2.078347632 |
| H | -6.9703111 | 6.94987494 | 1.673493275 |
| N | -4.0999774 | 6.75995892 | 0.521371113 |
| C | -5.0052664 | 6.1811094 | 1.381633133 |
| C | -3.5206253 | 5.76589618 | -0.126685175 |
| H | -2.7687339 | 5.85403198 | -0.914232097 |
| N | -3.9854974 | 4.56192233 | 0.288393078 |
| H | -3.8204564 | 3.63138018 | -0.146892494 |
| C | -4.944161 | 4.80623544 | 1.243596928 |
| H | -5.5411061 | 4.00483417 | 1.673851618 |
| C | 0.07760522 | 1.95171259 | -2.353453134 |
| H | 0.35614754 | 1.9164679 | -3.417467225 |
| C | -1.3030636 | 2.65060007 | -2.228152337 |
| H | -1.5247409 | 2.79070217 | -1.155139881 |
| H | -2.0821061 | 1.98248825 | -2.630917327 |
| N | -2.227343 | 4.10511292 | -4.081038647 |
| C | -1.430138 | 3.96406339 | -2.959900577 |
| C | -2.0684857 | 5.35250377 | -4.495846589 |
| H | -2.5360111 | 5.79119331 | -5.377202774 |
| N | -1.2207558 | 6.03965786 | -3.70110143 |
| H | -0.7883808 | 6.95247029 | -3.927717927 |
| C | -0.8121486 | 5.17534771 | -2.711512222 |
| H | -0.1388543 | 5.49393843 | -1.926969527 |
| C | 1.09201973 | 2.78041592 | -1.57125093 |
| O | 1.07162242 | 2.87708166 | -0.33928357 |
| N | 2.00930643 | 3.47849133 | -2.302806641 |
| H | 2.0348361 | 3.35140732 | -3.31483437 |
| C | 2.93510964 | 4.4056438 | -1.680460082 |
| H | 3.70552958 | 4.65149641 | -2.421942036 |
| C | 2.2624942 | 5.73231131 | -1.316645444 |
| H | 1.43037393 | 5.55063985 | -0.612892517 |
| H | 1.8560336 | 6.17532281 | -2.241597409 |
| O | 3.23164735 | 6.58047145 | -0.741401868 |
| H | 2.88954578 | 7.51515335 | -0.678680102 |
| C | 3.70070438 | 3.69808483 | -0.552142656 |
| O | 4.18271853 | 2.58278011 | -0.736407917 |
| N | 3.81578894 | 4.35496506 | 0.634902299 |
| H | 3.57154381 | 5.35121355 | 0.606256826 |
| C | 4.62299424 | 3.90065244 | 1.755674727 |
| H | 5.55922892 | 3.45450029 | 1.368896823 |
| C | 4.94469181 | 5.09097511 | 2.682339122 |
| H | 5.47539149 | 4.7478741 | 3.588641243 |
| H | 4.01084316 | 5.54317989 | 3.051077602 |
| N | 7.0137839 | 6.01538342 | 1.491640509 |
| H | 7.57853777 | 5.17278705 | 1.51879386 |
| C | 5.72450423 | 6.16200406 | 1.983241288 |
| C | 7.3548716 | 7.17916918 | 0.86307058 |
| H | 8.31723545 | 7.31546211 | 0.365775054 |
| N | 6.37967231 | 8.05944236 | 0.932143402 |
| C | 5.36683785 | 7.43977829 | 1.620114772 |
| H | 4.41897371 | 7.93940889 | 1.816104972 |
| C | 3.33420356 | 3.94858819 | -6.498890025 |
| H | 3.9757153 | 4.29226996 | -7.338545168 |
| C | 2.07661876 | 4.88511596 | -6.49586762 |
| H | 2.38867649 | 5.94125292 | -6.405796041 |
| H | 1.42244191 | 4.64131303 | -5.638166675 |
| C | 1.22094815 | 4.78469924 | -7.787665929 |
| O | 0.21835593 | 4.02583283 | -7.748110643 |
| O | 1.62247947 | 5.42344835 | -8.795622058 |
| C | -0.8264678 | 14.2186629 | -6.341379379 |
| H | -0.1167557 | 13.5883248 | -6.904818878 |
| C | -1.4062558 | 13.3915777 | -5.162103868 |
| H | -2.0479695 | 12.5601997 | -5.532004884 |
| H | -2.0815085 | 14.0113613 | -4.542117638 |
| C | -0.3889903 | 12.7590523 | -4.255462774 |
| C | 0.86035186 | 12.1966338 | -4.606616615 |
| H | 1.33143433 | 12.111054 | -5.584469621 |
| N | 1.49156739 | 11.7337257 | -3.501651721 |
| H | 2.37361344 | 11.1818773 | -3.508437717 |
| C | 0.68363776 | 11.9567846 | -2.384672899 |
| C | -0.5066492 | 12.585654 | -2.842826532 |
| C | -1.522127 | 12.8958356 | -1.911494523 |
| H | -2.458324 | 13.3442562 | -2.252496965 |
| C | -1.3114314 | 12.6160506 | -0.562267308 |
| H | -2.0760313 | 12.865868 | 0.175889142 |
| C | 0.91829508 | 11.6997105 | -1.043017478 |
| H | 1.85260953 | 11.2444876 | -0.716704688 |
| C | -0.0994476 | 12.0475798 | -0.130528477 |
| H | 0.06773706 | 11.8966786 | 0.939254692 |
| C | -0.6803411 | 10.239486 | -8.918156098 |
| H | -0.0507968 | 9.73159136 | -9.672101512 |
| C | 0.23072206 | 11.036545 | -7.967531479 |
| H | 1.02342604 | 10.4263738 | -7.496377264 |
| H | -0.342365 | 11.5114992 | -7.150587638 |
| H | 0.74020571 | 11.8457729 | -8.518325569 |
| N | 0.12500718 | 9.30121709 | 3.030697181 |
| H | -0.4474105 | 9.19468957 | 3.894453713 |
| C | -0.3336072 | 8.68163597 | 1.920957022 |
| C | -1.6831206 | 8.03635097 | 1.973477499 |
| H | -2.3465665 | 8.34463205 | 1.14727919 |
| H | -1.5900494 | 6.93760198 | 1.90150529 |
| H | -2.2000426 | 8.26188284 | 2.916738594 |
| C | 0.46704826 | 8.61437288 | 0.780059913 |
| O | 0.07074191 | 7.96283158 | -0.342853924 |
| H | -0.8261191 | 7.6009079 | -0.232452308 |
| C | 1.79219349 | 9.1807708 | 0.758098202 |
| O | 2.55193626 | 9.05170189 | -0.239206587 |
| C | 2.19307985 | 9.91200899 | 1.950166147 |
| C | 3.43608505 | 10.5868625 | 2.009081947 |
| H | 4.07050762 | 10.5779665 | 1.119881059 |
| C | 3.84004534 | 11.2249036 | 3.170514241 |
| H | 4.79525009 | 11.7578464 | 3.204401544 |
| C | 3.01222664 | 11.1790921 | 4.314308231 |
| H | 3.34200093 | 11.6225697 | 5.255182921 |
| C | 1.77455226 | 10.5573995 | 4.277907444 |
| H | 1.12409414 | 10.5650369 | 5.157275951 |
| C | 1.34948581 | 9.92536639 | 3.091192346 |
| O | 1.92659928 | 6.61696922 | 3.276209594 |
| O | 1.40971828 | 6.00915308 | 2.378854952 |
| H | -1.4445777 | 5.35301534 | 4.403032078 |
| H | -6.4331243 | 5.70191652 | 3.913778321 |
| H | 0.05689578 | 0.95220487 | -1.919145916 |
| H | 4.16917112 | 3.074132 | 2.302461575 |
| H | 3.04014252 | 2.9222499 | -6.718492245 |
| H | 3.94135316 | 4.17183213 | -5.621622204 |
| H | -0.289958 | 15.1119555 | -6.021609186 |
| H | -1.6199496 | 14.3805509 | -7.070931432 |
| H | -1.3571805 | 10.9290567 | -9.422584329 |
| H | -1.1249188 | 9.42790662 | -8.342172564 |

Cartesian coordinates (in Å) for **H251A QM region at 500 ps**:

| N | -0.7986243 | 5.70328454 | 5.222013918 |
| --- | --- | --- | --- |
| H | 0.06728499 | 5.14485923 | 5.266958792 |
| C | -1.1026154 | 6.56077921 | 6.37743806 |
| H | -1.6157787 | 6.00390017 | 7.182787617 |
| C | 0.20581405 | 7.12994908 | 6.980051979 |
| H | -0.1105979 | 7.95639661 | 7.643240723 |
| H | 0.79071357 | 7.57758458 | 6.157950399 |
| C | 1.04948096 | 6.1789156 | 7.771351156 |
| C | 2.32253912 | 5.73400563 | 7.458306873 |
| H | 2.92548021 | 5.94489755 | 6.574936076 |
| N | 2.79389881 | 4.9021364 | 8.440353753 |
| H | 3.57211634 | 4.26275631 | 8.293232039 |
| C | 1.82676877 | 4.76013305 | 9.418995846 |
| C | 0.71912697 | 5.57200805 | 9.039196002 |
| C | -0.4049056 | 5.6361553 | 9.886849105 |
| H | -1.2528145 | 6.27938493 | 9.634778175 |
| C | -0.4164977 | 4.86717927 | 11.05023475 |
| H | -1.2888105 | 4.90819272 | 11.71039048 |
| C | 1.82825326 | 4.0075258 | 10.59809893 |
| H | 2.67672865 | 3.38029848 | 10.88266768 |
| C | 0.68697929 | 4.0588295 | 11.40256849 |
| H | 0.66644307 | 3.46988513 | 12.32425458 |
| C | -1.9897037 | 7.7892091 | 6.017763799 |
| O | -1.6321353 | 8.6513599 | 5.209022796 |
| N | -3.1825249 | 7.79589675 | 6.63532183 |
| H | -3.3941692 | 7.00876365 | 7.269080092 |
| C | -4.2833787 | 8.71280003 | 6.363139932 |
| H | -4.9442921 | 8.62080044 | 7.239298807 |
| C | -3.9007432 | 10.1905725 | 6.242723455 |
| H | -4.8367955 | 10.7636761 | 6.229443976 |
| H | -3.3475871 | 10.3799384 | 5.314481934 |
| S | -2.8813318 | 10.6882285 | 7.676656641 |
| H | -3.0231072 | 12.0299454 | 7.511579059 |
| C | -5.1990278 | 8.33170205 | 5.175168188 |
| O | -6.0698671 | 9.12850469 | 4.859839689 |
| N | -5.0852818 | 7.09770359 | 4.606401578 |
| H | -4.2866371 | 6.44035239 | 4.769965056 |
| C | -6.1688498 | 6.64312183 | 3.760079475 |
| H | -7.0303005 | 7.28106513 | 4.020661958 |
| C | -6.012087 | 6.75068095 | 2.231352 |
| H | -5.8233429 | 7.80666287 | 2.007782809 |
| H | -7.0007687 | 6.55512868 | 1.770580375 |
| N | -4.1438895 | 6.48572563 | 0.567583319 |
| C | -4.9799935 | 5.92495011 | 1.507226514 |
| C | -3.4818548 | 5.48355027 | 0.019354866 |
| H | -2.7569146 | 5.55922627 | -0.793572372 |
| N | -3.8296167 | 4.29223527 | 0.565166227 |
| H | -3.4932632 | 3.34983958 | 0.284833296 |
| C | -4.7939473 | 4.55164184 | 1.513838581 |
| H | -5.3296564 | 3.75361115 | 2.02579244 |
| C | 0.10317905 | 1.94416502 | -2.314352588 |
| H | 0.43542441 | 1.8299523 | -3.358953053 |
| C | -1.174486 | 2.84132537 | -2.294203494 |
| H | -1.4289705 | 3.06045505 | -1.244736748 |
| H | -2.0195019 | 2.28711575 | -2.738195481 |
| N | -1.103772 | 4.19689504 | -4.411954916 |
| C | -1.0128878 | 4.1386104 | -3.040251691 |
| C | -0.8167497 | 5.43680876 | -4.763834417 |
| H | -0.761658 | 5.8244364 | -5.782478374 |
| N | -0.5575455 | 6.20294009 | -3.680404833 |
| H | -0.2356105 | 7.17865337 | -3.738736892 |
| C | -0.6744861 | 5.39538574 | -2.571925551 |
| H | -0.4585819 | 5.75461953 | -1.572241012 |
| C | 1.15084454 | 2.6934926 | -1.501057774 |
| O | 1.05076466 | 2.86424258 | -0.278778777 |
| N | 2.18100335 | 3.24637411 | -2.196642783 |
| H | 2.2899384 | 3.02313428 | -3.188142864 |
| C | 3.11204468 | 4.15170964 | -1.558527256 |
| H | 3.95317205 | 4.30891318 | -2.247425114 |
| C | 2.45904146 | 5.52167135 | -1.340364365 |
| H | 1.59371284 | 5.41528564 | -0.666008548 |
| H | 2.09851302 | 5.88000787 | -2.319942241 |
| O | 3.41786037 | 6.4076646 | -0.798403244 |
| H | 3.01771324 | 7.30550027 | -0.650043475 |
| C | 3.72726471 | 3.46927116 | -0.326372214 |
| O | 4.10148574 | 2.30033764 | -0.374227136 |
| N | 3.8333658 | 4.22375865 | 0.803812927 |
| H | 3.68244012 | 5.22948646 | 0.664172102 |
| C | 4.54206533 | 3.81447659 | 2.005631095 |
| H | 5.47297919 | 3.28987289 | 1.716689988 |
| C | 4.8771424 | 5.05629081 | 2.85878839 |
| H | 5.39367849 | 4.75821035 | 3.787788607 |
| H | 3.9488997 | 5.55496455 | 3.182963544 |
| N | 6.99112361 | 5.89388435 | 1.675688843 |
| H | 7.5755167 | 5.07646941 | 1.81943249 |
| C | 5.68308576 | 6.06477691 | 2.100381651 |
| C | 7.33120614 | 6.9823569 | 0.924310887 |
| H | 8.31238767 | 7.08923285 | 0.459370853 |
| N | 6.32766997 | 7.83408683 | 0.840764566 |
| C | 5.30764504 | 7.27827092 | 1.572024978 |
| H | 4.33891581 | 7.76649331 | 1.661229554 |
| C | 3.75120268 | 3.58966245 | -6.264693397 |
| H | 4.36229217 | 3.95538764 | -7.114346412 |
| C | 2.42760855 | 4.42334279 | -6.260993106 |
| H | 2.65104273 | 5.50178094 | -6.161366731 |
| H | 1.78268977 | 4.12640974 | -5.41450961 |
| C | 1.60664555 | 4.27218573 | -7.560321269 |
| O | 0.61497167 | 3.49851539 | -7.55303892 |
| O | 2.00470989 | 4.89738469 | -8.578419787 |
| C | -0.523068 | 13.2024544 | -6.101282162 |
| H | 0.13692867 | 12.4207675 | -6.51516535 |
| C | -1.1427284 | 12.6722247 | -4.784687201 |
| H | -1.767634 | 11.7739894 | -4.970706665 |
| H | -1.844999 | 13.4138225 | -4.351496087 |
| C | -0.142932 | 12.3244247 | -3.710701835 |
| C | 1.25568827 | 12.2071 | -3.83504 |
| H | 1.87027954 | 12.2657687 | -4.731664185 |
| N | 1.82608703 | 12.0252728 | -2.623030811 |
| H | 2.84064891 | 11.9954958 | -2.450433657 |
| C | 0.83626582 | 11.992618 | -1.650356784 |
| C | -0.4125415 | 12.1698855 | -2.312731954 |
| C | -1.5983278 | 12.1688154 | -1.556780101 |
| H | -2.5616819 | 12.2933029 | -2.055690107 |
| C | -1.5207579 | 11.9898697 | -0.17238312 |
| H | -2.4348374 | 11.9732809 | 0.428107477 |
| C | 0.92733075 | 11.8347876 | -0.274839408 |
| H | 1.89140473 | 11.7298787 | 0.224626713 |
| C | -0.273397 | 11.8286393 | 0.460157778 |
| H | -0.229912 | 11.7178711 | 1.547257544 |
| C | -0.4550197 | 9.57154785 | -9.225674813 |
| H | -0.0135419 | 8.88092826 | -9.963891415 |
| C | 0.67965608 | 10.4106864 | -8.606731265 |
| H | 0.32577256 | 11.0844866 | -7.809247 |
| H | 1.13927779 | 11.0556152 | -9.378869383 |
| H | 1.50134531 | 9.80540895 | -8.182154721 |
| N | 0.04036115 | 9.12566791 | 3.1288331 |
| H | -0.5558653 | 9.04059582 | 3.980313699 |
| C | -0.3308711 | 8.41527174 | 2.042765748 |
| C | -1.6393951 | 7.68635905 | 2.089912311 |
| H | -2.3122998 | 7.94254832 | 1.252200193 |
| H | -1.4757533 | 6.5939561 | 2.040385064 |
| H | -2.1842127 | 7.90262402 | 3.020093801 |
| C | 0.50981154 | 8.34938792 | 0.935107893 |
| O | 0.16276213 | 7.65199697 | -0.179385505 |
| H | -0.7568026 | 7.34461907 | -0.104589674 |
| C | 1.81785147 | 8.95695504 | 0.936931519 |
| O | 2.62285873 | 8.81311765 | -0.018811286 |
| C | 2.13941616 | 9.74986122 | 2.119553815 |
| C | 3.35121873 | 10.4755433 | 2.212268492 |
| H | 4.05558236 | 10.4000901 | 1.380596064 |
| C | 3.62921974 | 11.2570302 | 3.322642833 |
| H | 4.5624896 | 11.8269485 | 3.382511665 |
| C | 2.69347416 | 11.3255645 | 4.380692793 |
| H | 2.8859307 | 11.9284084 | 5.268488332 |
| C | 1.50287244 | 10.6240586 | 4.322060999 |
| H | 0.77557651 | 10.6956491 | 5.136258986 |
| C | 1.21773429 | 9.82619875 | 3.194658843 |
| O | 1.63647769 | 6.1538268 | 3.056165133 |
| O | 1.2168022 | 5.26107632 | 2.369932509 |
| H | -1.5512735 | 5.24686693 | 4.74748807 |
| H | -6.4296777 | 5.63134577 | 4.070474886 |
| H | -0.0340324 | 0.97552737 | -1.833747308 |
| H | 4.01502499 | 3.06415287 | 2.594961328 |
| H | 3.54087041 | 2.53495673 | -6.441960138 |
| H | 4.35359974 | 3.85875745 | -5.397068489 |
| H | 0.08163627 | 14.0944131 | -5.93754525 |
| H | -1.2895772 | 13.2957763 | -6.870581818 |
| H | -1.1653189 | 10.2248395 | -9.732382199 |
| H | -0.8885362 | 8.91518142 | -8.47114588 |
|  |  |  |  |
|  |  |  |  |
